# Identification of Transcriptomic Differences in Induced Pluripotent Stem Cells and Neural Progenitors from Amyotrophic Lateral Sclerosis Patients Carrying Different Mutations: A Pilot Study

**DOI:** 10.3390/cells14130958

**Published:** 2025-06-23

**Authors:** Chiara Sgromo, Martina Tosi, Cristina Olgasi, Fabiola De Marchi, Francesco Favero, Giorgia Venturin, Beatrice Piola, Alessia Cucci, Lucia Corrado, Letizia Mazzini, Sandra D’Alfonso, Antonia Follenzi

**Affiliations:** 1 Department of Health Sciences, Università degli Studi del Piemonte Orientale, 28100 Novara, Italy; chiara.sgromo@uniupo.it (C.S.); martina.tosi@uniupo.it (M.T.); 20026223@studenti.uniupo.it (G.V.); beatrice.piola@uniupo.it (B.P.); alessia.cucci@uniupo.it (A.C.); lucia.corrado@uniupo.it (L.C.); sandra.dalfonso@uniupo.it (S.D.); antonia.follenzi@uniupo.it (A.F.); 2 Department of Translational Medicine, Università degli Studi del Piemonte Orientale, 28100 Novara, Italy; cristina.olgasi@med.uniupo.it (C.O.); francesco.favero@med.uniupo.it (F.F.); 3 ALS Centre, Neurology Unit, Department of Translational Medicine, Maggiore della Carità Hospital, Università degli Studi del Piemonte Orientale, 28100 Novara, Italy; fabiola.demarchi@uniupo.it; 4 CAAD (Center on Autoimmune and Allergic Diseases), Università degli Studi del Piemonte Orientale, 28100 Novara, Italy; 5 Dipartimento Attività Integrate Ricerca Innovazione, Azienda Ospedaliero-Universitaria SS. Antonio e Biagio e C. Arrigo, 15122 Alessandria, Italy

**Keywords:** Amyotrophic lateral sclerosis, induced pluripotent stem cells, transcriptomic analysis, RNA-seq, neural progenitor cells

## Abstract

Amyotrophic lateral sclerosis (ALS) is a devastating neurodegenerative disease affecting motor neurons with a phenotypic and genetic heterogeneity and elusive molecular mechanisms. With the present pilot study, we investigated different genetic mutations (*C9orf72*, *TARDBP*, and *KIF5A*) associated with ALS by generating induced pluripotent stem cells (iPSCs) from peripheral blood of ALS patients and healthy donors. iPSCs showed the typical morphology, expressed stem cell markers both at RNA (*OCT4*, *SOX2*, *KLF4*, and *c-Myc*) and protein (Oct4, Sox2, SSEA3, and Tra1-60) levels. Moreover, embryoid bodies expressing the three germ-layer markers and neurospheres expressing neural progenitor markers were generated. Importantly, the transcriptomic profiles of iPSCs and neurospheres were analyzed to highlight the differences between ALS patients and healthy controls. Interestingly, the differentially expressed genes (DEGs) shared across all ALS iPSCs are linked to extracellular matrix, highlighting its importance in ALS progression. In contrast, ALS neurospheres displayed widespread deficits in neuronal pathways, although these DEGs were varied among patients, reflecting the disease’s heterogeneity. Overall, we generated iPSC lines from ALS patients with diverse genetic backgrounds offering a tool for unravelling the intricate molecular landscape of ALS, paving the way for identifying key pathways implicated in pathogenesis and the disease’s phenotypic variability.

## 1. Introduction

Amyotrophic lateral sclerosis (ALS) is a fatal neurodegenerative disease that affects upper and lower motor neurons (MNs), leading to the loss of voluntary muscle control, muscle weakness, and eventual paralysis. ALS is predominantly a multifactorial disorder characterized by great phenotypic heterogeneity, cognitive dysfunction, and behavioral changes. Indeed, there are two main forms of ALS, according to the disease’s family history: sporadic ALS (sALS), which represents 85–90% of cases, occurring without a known family history, and familial ALS (fALS), which accounts for 10–15% of cases, where a positive family history of ALS is reported [1]. Multiple factors contribute to the development of ALS, with a significant genetic component being identified. Pathogenic sequence variations in several genes have been strongly associated with the disease. In particular, mutations in *SOD1*, *C9orf72*, *FUS*, and *TARDBP* genes account for about 48% of fALS and about 5% of sALS cases within populations of European origin [2]. The GGGGCC hexanucleotide repeat expansion in the first intron of *C9orf72* is the most common genetic cause of ALS accounting for about 40% of fALS. This mutation causes an abnormal production of RNAs that produce toxic dipeptide repeat protein (DPR) accumulation, impairing nucleocytoplasmic transport, nucleotide metabolism, lysosomal processes, and cellular metabolic pathways [3]. *C9orf72* (chromosome 9 open reading frame 72) regulates autophagy and vesicular trafficking in neurons, and it is involved in actin dynamics and endosomal recycling at synapses [4]. The TARDBP gene encodes the transactive response (TAR) DNA-binding protein 43 (TDP-43), which plays a crucial role in RNA metabolism. To date, approximately 40 different *TARDBP* missense variants have been identified. A mutated form of TDP-43 binds to ubiquitin and forms aggregates, leading to the formation of inclusions found in the MNs and brains of ALS patients [5]. More recently, thanks to next-generation sequencing approaches, over 40 new ALS genes have been identified, accounting for a rarer proportion of ALS cases. Among these, a recent study identified *KIF5A* as a novel ALS gene through rare variant burden analysis [6]. *KIF5A* encodes a member of the kinesin family of proteins that functions as a microtubule motor, necessary for organelle trafficking, cell division, and axonal maintenance. Genetic analyses have identified multiple loss-of-function mutations in ALS: these include frameshift and splice-site mutations that lead to the skipping of *KIF5A* exon 27 and the production of a truncated protein with impaired cargo-binding function and axonal transport disruption [6,7]. Although the different ALS-related mutations affect distinct molecular pathways, all of them lead to the common outcome: MN degeneration. Currently, there is no effective treatment to halt or reverse ALS progression, and patients are treated to slow down disease progression and alleviate symptoms. Therefore, the urgent need for more effective treatments and a deeper understanding of the molecular mechanisms underlying ALS has driven the use of cellular models such as the induced pluripotent stem cells (iPSCs) [8,9,10]. In this pilot study, we generated iPSCs by reprogramming CD34+ cells isolated from peripheral blood of four ALS patients carrying different mutations. iPSCs were further differentiated into neurospheres, a critical intermediate stage in MN differentiation, characterized by self-renewal, multipotent differentiation, and ability to closely replicate early neurodevelopmental processes. Transcriptomic analysis highlighted common transcriptomic signatures and disease-related enriched pathways in ALS iPSCs. On the other hand, transcriptomic analysis of neurospheres revealed a modulation of neural-specific pathways, thereby highlighting a dysregulation of the neural function already at the stage of progenitor cells. In conclusion, these findings could provide the molecular basis for ALS modeling and potentially reveal key transcriptomic insights into distinct mutations.

## 2. Materials and Methods

### 2.1. Patients

Three healthy donors and four patients diagnosed with ALS and carrying three different heterozygous mutations *(KIF5A*, *C9orf72*, and *TARDBP*) were recruited at the Expert ALS Center of the Maggiore della Carità University Hospital, Novara, Italy. According to El Escorial Criteria—Revised [11,12], we included only patients classified as “definite ALS”. Demographic and clinical information of ALS patients are reported in Table 1. All enrolled subjects provided written informed consent to participate in the study, and blood samples were collected upon genetic test result notice (within six months of clinical diagnosis). The study has been approved by the ethical committee of the Maggiore della Carità Hospital (protocol 1102/CE, study number n. CE 233/20). In addition to genetic analysis, for demographic and clinical variables, for each included patient, we considered gender, age at onset, positive family history for ALS, cognitive status, disease phenotype (prevalent upper or lower MNs; spinal/bulbar), disease duration from symptoms onset to death, and cognitive status [13]. 

### 2.2. CD34+ Isolation

Mononuclear cells were purified by gradient centrifugation on Ficoll (GE Healthcare, Chicago, IL, USA), and CD34+ cells were isolated by using MACS MicroBeadKit (Miltenyi Biotec, Bergisch Gladbach, Germany) according to the manufacturer’s protocol. Isolated cells were cultured for 3 days in StemPro34 medium (Gibco, ThermoFisher, Waltham, MA, USA) supplemented by 100 ng/mL of hSCF, 50 ng/mL of IL-3, and 25 ng/mL of GM-CSF (Immunotools, Friesoythe, Germany) [14].

### 2.3. iPSCs Reprogramming

Four days after isolation, 9 × 10^4^ cells were transduced with a Sendai reprogramming vectors kit (ThermoFisher, Waltham, MA, USA) at different multiplicities of infection following the manufacturer’s instructions. After three days, transduced cells were plated on a layer of recombinant Vitronectin (ThermoFisher, Waltham, MA, USA) with StemPRO34 medium without cytokines. Gradually, medium was replaced with Essential8 medium (GibcoThermoFisher, Waltham, MA, USA) to maintain iPSCs in culture.

### 2.4. iPSC Culture

After 15/20 days after reprogramming, iPSCs appeared, and a clonal selection was performed. The single clones were manually picked up and transferred to a well of a 24 vitronectin-coated plate. For iPSC culture and clone stabilization, several splitting ratios were used. Cells were kept in culture with Essential8 medium (Gibco), and medium was changed every day.

### 2.5. Alkaline Phosphatase Staining

Alkaline phosphatase (AP) was performed as previously described [14]. Briefly, iPSCs were fixed with 4% of PAF and stained using the AP detection kit (Millipore, Burlington, MA, USA) according to the manufacturer’s protocol. 

### 2.6. RNA Isolation and RT-PCR

RNA extraction was performed using Trizol reagent (Invitrogen, Carlsbad, CA, USA). A total of 1 μg of total RNA was reverse transcribed with a RevertAid First Strand cDNA Synthesis Kit (Thermo Scientific, Waltham, MA, USA), and the cDNA was used to perform PCRs. All the PCRs were performed with Platinum™ Taq DNA Polymerase (Invitrogen). Primers, annealing temperatures, and product sizes are listed below. PCR products were resolved in 2% agarose gels. HEK293T cells (cells exhibiting epithelial morphology isolated from human embryo kidney tissue, ATCC# CRL-11268) transduced with the Sendai virus were used as positive control. Primers used are listed in Supplementary Table S2.

### 2.7. Immunofluorescence Staining

iPSCs were cultured on cover glass in a 24-well plate. Cells were fixed in cold PFA 4% for 10 min, permeabilized with 0.5% PBS-TritonX100, and incubated with blocking buffer (5% goat serum, 1% BSA, 0.1% Triton X-100 in PBS) for 1 h at room temperature (RT). Primary antibodies were used with the correct dilution in 5% goat serum, 1% BSA, and 0.1% Triton X-100 in PBS and incubated for 2 h at RT. Secondary antibodies were prepared in 0.5% PBS-TritonX100 and incubated for 45 min at RT. Nuclei were stained with DAPI (Sigma, Burlington, MA, USA). Primary and secondary antibodies with the dilutions are detailed in Supplementary Table S3.

### 2.8. Embryoid Body Generation

When all iPSC lines showed stable morphology without any type of differentiated cells and 85% of confluence was reached, they were detached and plated in a low-adhesion well with Essential8 for 5 days. 

### 2.9. Neurosphere Generation

Healthy and ALS iPSCs were detached when they reached 80% of confluence and dissociated, and 15 × 10^4^ cells were plated with Essential 8 and 10 µM of Rock Inhibitor (Y-27632) (Sigma-Aldrich, Burlington, MA, USA). The induction of neurosphere differentiation was achieved using medium composed of DMEM/F12 (Gibco), neurobasal medium (Gibco) 0.5× N2 (Gibco), 0.5× B27 (Gibco), Glutamax (Gibco), and non-essential amino acids (Gibco) supplemented by small molecules such as SB431542 (Stem Cell Technologies, Vancouver, BC, Canada), LDN193189 (Stem Cell Technologies), CHIR99021 (Stem Cell Technologies), ascorbic acid (Sigma), all-trans RA (Stem Cell Technologies), and purmorphamine (Stem Cell Technologies). For neurosphere generation we followed the main steps described in the literature [15,16].

### 2.10. Western Blot

iPSCs were lysed in RIPA buffer supplemented with 100× protease inhibitors (Thermo Fisher Scientific). Protein concentration was determined using the BCA Protein Assay Kit (Thermo Fisher Scientific). Equal amounts of protein samples (20 µg) were resolved by SDS-PAGE using different gel concentrations depending on the target protein. Membranes were incubated overnight at 4 °C with the following primary antibodies: Anti-TDP-43 (1:1000), Anti-C9orf72 (1:1000), Anti-KIF5A (1:1000), Anti-vinculin (1:3000, SantaCruz, Dallas, TX, USA). Proteins were visualized using enhanced chemiluminescence (ECL) (Bio-Rad, Hercules, CA, USA) and detected using a ChemiDoc imaging system (Bio-Rad). MDA-MB-231 cells (human breast cancer cell line, ATCC# HTB-26) and SH-SY5Y (human neuroblastoma cell line, ATCC#CRL-2266) were used as positive controls for the proteins tested in the WB analysis.

### 2.11. Mutational Analysis of iPSCs

To confirm the occurrence of each of the mutations identified in the ALS patients from which iPSCs have been derived, we performed Sanger sequencing on ABI PRISM 3130XL for the mutations in *KIF5A* and *TARDBP* genes and PCR-tandem repeat and analysis on ABI PRISM 3130XL for the *C9orf72* pathogenetic GGGGCC tandem repeat expansion. For each patient, we confirmed the heterozygous mutation originally identified in PBMC from peripheral blood in the corresponding iPSCs utilized in this study (Supplementary Figure S1).

### 2.12. RNA Sequencing

The transcriptome of iPSCs and neurospheres derived from four male patients with pathogenetic sequence variations in three different ALS genes, namely *KIF5A* (exon 27 3′ splice junction variant, c.3020+1G>A, NM_004984: chr12:57582630G>A, hg38, ALS_001), *TARDBP* (p.Ala382Thr, c.1144G>A, NM_007375.4, chr1-11022553G>A, hg38 ALS_002 and ALS_004), and *C9orf72* (intron 1 GGGGCC repeat pathogenetic expansion, ALS_003). Two gender-matched healthy controls (H001 and H003) were included in the study. For each subject, a technical duplicate was obtained from iPSCs at passages p17-p25 and from neurospheres, resulting in a total of 24 samples. 

RNA extraction was performed using the miRNeasy Micro Kit (QIAGEN, Venlo, The Netherlands) according to the manufacturer’s instructions. For each sample, 500 ng of RNA were used for library preparation with the Illumina Stranded Total RNA Prep with the Ribo-Zero Plus kit. The first 10 samples (iPSCs of the four patients and the first healthy control) were sequenced on Illumina NextSeq 550 sequencer (Illumina, San Diego, CA, USA), while the remaining 14 samples (iPSCs of the second control and all the neurospheres) were sequenced on a Novaseq sequencer (Illumina, San Diego, CA, USA). Initial quality checks on reads were assured using FastQC [17]: the average phred score quality for each base was ≥30; therefore, reads have not been trimmed. An RSEM computational pipeline [18] was used to quantify gene expression levels using the STAR aligner [19]. GRChg38/hg38, annotated with the Ensembl v113 database, was used as the reference genome. Only genes with a TPM > 1 in at least one sample were considered to be expressed and underwent further analysis, after batch-effect correction. Statistical and graphical computations were performed in the R environment (www.r-project.org, accessed on 14 March 2025). PCA (principal component analysis) plotting was performed using the “prcomp” package of R on the matrix of expression data. Differentially expressed genes (DEGs) were calculated using DESeq2 [20] with |log2FC| > 2 and *p*.adj ≤ 0.05 as parameters to define the statistical significance of differential gene expression. Enrichment analysis was conducted with Metascape [21] (min overlap = 3; pval cut-off = 0.01; min enrichment = 1.5). 

### 2.13. Data Availability

Anonymized data and RNA-seq supporting the findings of the study will be made available by the corresponding author, upon reasonable request, for any qualified investigator. 

## 3. Results

### 3.1. Generation of iPSCs from CD34+ Cells Isolated from ALS Patients and Healthy Donors

**Figure 1:**
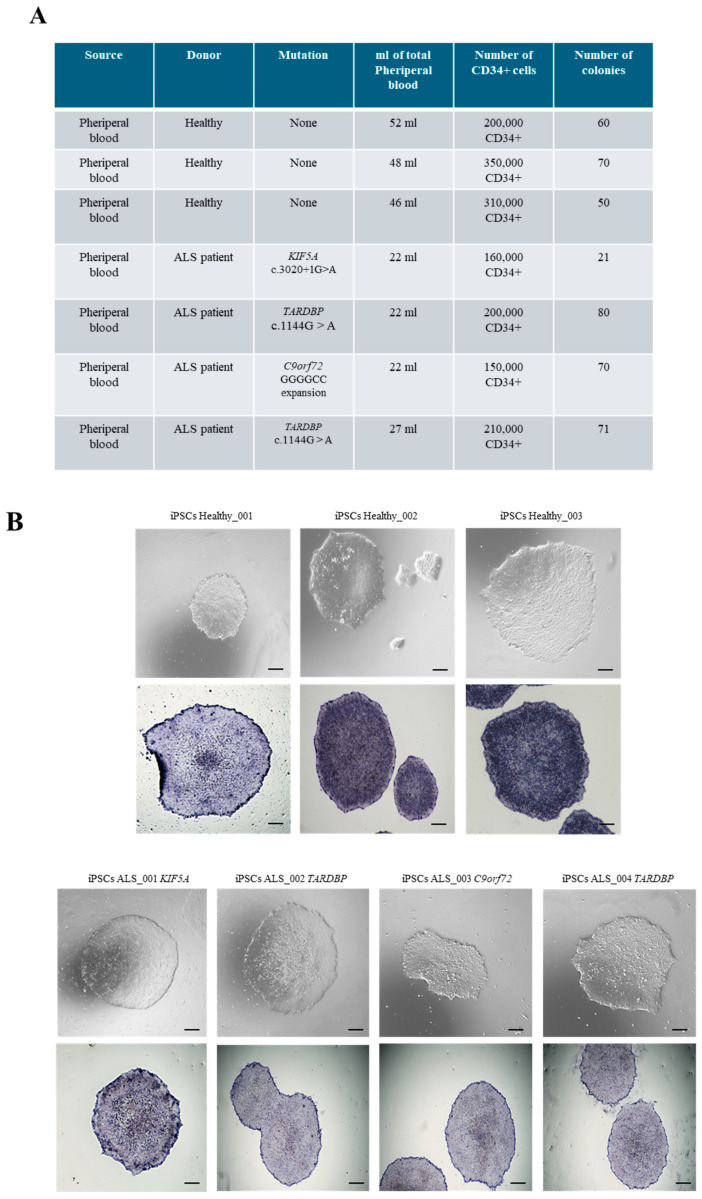
CD34+ cells reprogramming into iPSCs. (**A**) List of ALS and healthy donors from whom iPSCs were generated. (**B**) Upper panel: Representative phase-contrast microscopy showing ESC-like morphology of both healthy and ALS CD34+ cell-derived iPSCs. Lower panel: Positivity for alkaline phosphatase staining of healthy and ALS iPSCs. Scale bars, 100 μm.

After informed consent, blood samples from four fALS patients (consistent with autosomal dominance inheritance and defining ALS based on El Escorial revised criteria) [11,12] and three healthy donors were collected to generate ALS and healthy iPSCs (Table 1). In detail, we isolated CD34+ cells from non-mobilized peripheral blood of four fALS patients carrying different mutations and from three healthy donors, and we reprogrammed them with the Sendai virus. The main demographic and clinical features are summarized in Table 1. We obtained a comparable number of iPSC colonies among healthy and ALS reprogrammed CD34+ cells (Figure 1A). An exception was observed in ALS patients harboring the *KIF5A* mutation, where a noticeable reduction in the number of isolated CD34+ cells and colonies was detected (Figure 1A). The efficiency of the reprogramming process was consistent among the different samples, leading to the generation of iPSC lines from both healthy donors and ALS patients (Figure 1B). Indeed, iPSCs showed the typical ESC-like morphology, with compact colonies with well-defined borders, and were positive for alkaline phosphatase (AP) (Figure 1B). Furthermore, we sequenced ALS iPSC lines to assess genetic stability. Notably, even the mutation involving the hexanucleotide repeat expansion (G4C2) in *C9orf72* was preserved in iPSCs, confirming that the reprogramming process did not alter the genetic features (Supplementary Figure S1).

### 3.2. Characterization of iPSCs from CD34+ Cells Isolated from ALS Patients and Healthy Donors

**Figure 2:**
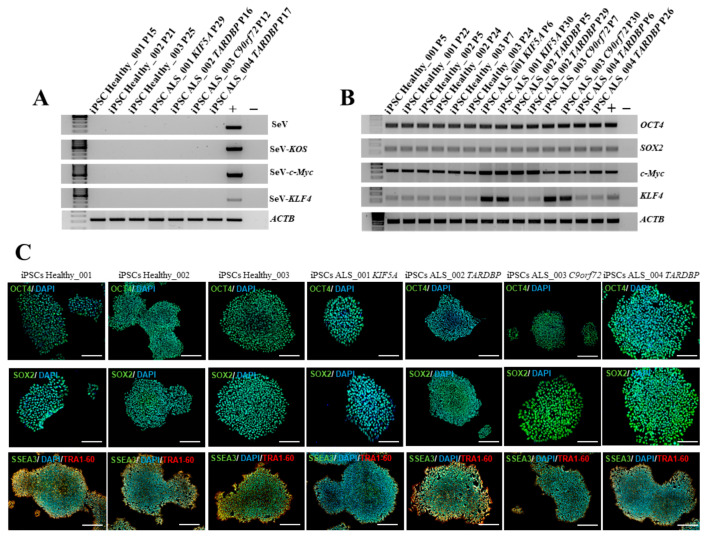

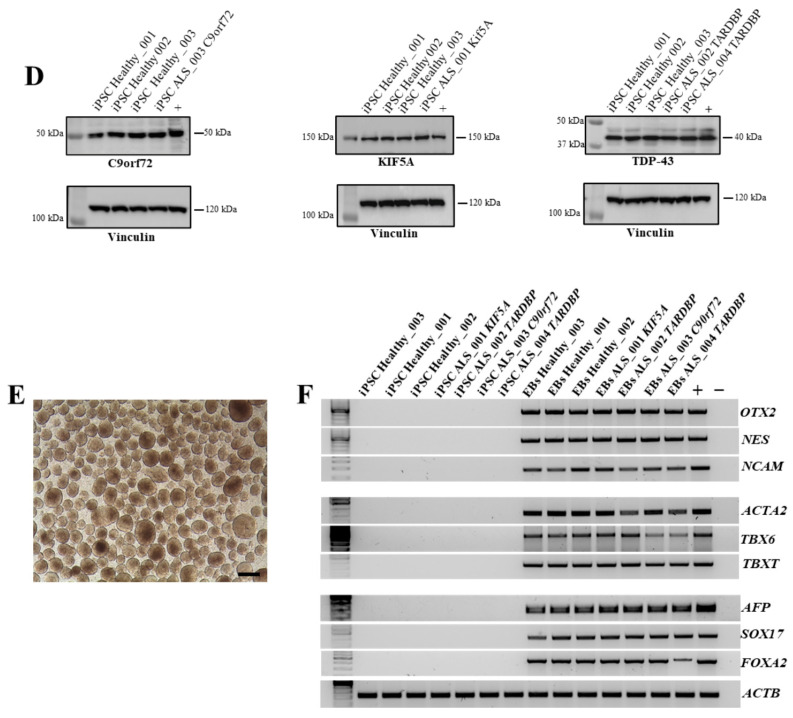
Characterization of healthy and ALS iPSCs. (**A**) RT-PCR for exogenous factors on healthy and ALS iPSCs. iPSCs from a healthy donor 15 days after reprogramming with Sendai virus was used as positive control. (**B**) RT-PCR for endogenous factors (*OCT4*, *SOX2*, *c-Myc*, and *KLF4*) on healthy and ALS iPSCs. HEK293T cells transduced with the Sendai virus were used as positive control. (**C**) Stem-cell marker expression at the protein level was detected by immunofluorescence on healthy and ALS iPSCs. Markers used were: OCT4 (green), SOX2 (green), SSEA3 (green), TRA1-60 (red), and DAPI (blue). Scale bars, 200 μm. (**D**) Western blot analysis of C9orf72, KIF5A, and TDP-43 expression. Positive controls: C9orf72: SH-SY5Y cell line; KIF5A: SH-SY5Y cell line; TDP-43: MDA-MB-231 cell line. (**E**) Representative image of EB generated from the culture of iPSCs in low adhesion plate. Scale bar, 100 μm. (**F**) RT-PCR for the three germ-layer markers on iPSCs-derived EBs: *OTX2*, *NCAM*, and *NES* (ectoderm); *ACTA2*, *TBX6*, and *TBXT* (mesoderm); *AFP*, *SOX17*, and *FOXA2* (endoderm). All data are representative of three independent experiments.

All iPSC clones were characterized by the expression of exogenous and endogenous pluripotency markers. Considering the nature of the Sendai Virus, the residual amount of virus in obtained healthy and ALS iPSCs were evaluated. Specifically, we selected iPSCs that had reached the 10th passage, and by RT-PCR we demonstrated that there was no residual of the Sendai virus in transduced cells, confirming the silencing of exogenous markers compared to positive controls represented by iPSCs 15 days post-reprogramming (Figure 2A). On the contrary, endogenous markers were highly expressed at the RNA level by iPSCs at the same passages (Figure 2B), demonstrating the stable reprogramming of iPSCs and their sustained staminality. These data were also demonstrated at the protein level, showing a high expression of Oct4, Sox2, Ssea3, and Tra1-60 both in healthy and ALS iPSCs (Figure 2C). To further confirm the expression of ALS-associated mutated genes in the iPSC lines, we assessed protein levels for TARDBP, KIF5A, and C9orf72. As shown in Figure 2D, C9orf72, KIF5A, and TDP-43, were detected at protein levels in iPSCs. These results demonstrate that the ALS-related genes are expressed at both transcriptional and protein levels in healthy and ALS iPSC lines. Finally, we exploited the ability of iPSCs to mimic embryogenesis at the level of the gastrula by generating embryoid bodies (EBs) from both healthy and ALS iPSCs (Figure 2E). We investigated the expression of key markers for the three germ layers by RT-PCR: *NES*, *NCAM*, and *OTX2* for the ectoderm; *ACTA2*, *TBXT*, and *TBX6* for the mesoderm; and *FOXA2*, *SOX17*, and *AFP* for the endoderm (Figure 2F). Our results demonstrated that all iPSC lines successfully generated EBs that expressed high levels of the typical markers of ectoderm, mesoderm, and endoderm, confirming the differentiation potential of the obtained iPSCs. 

### 3.3. Neurosphere Generation and Neuroectoderm Marker Expression

**Figure 3:**
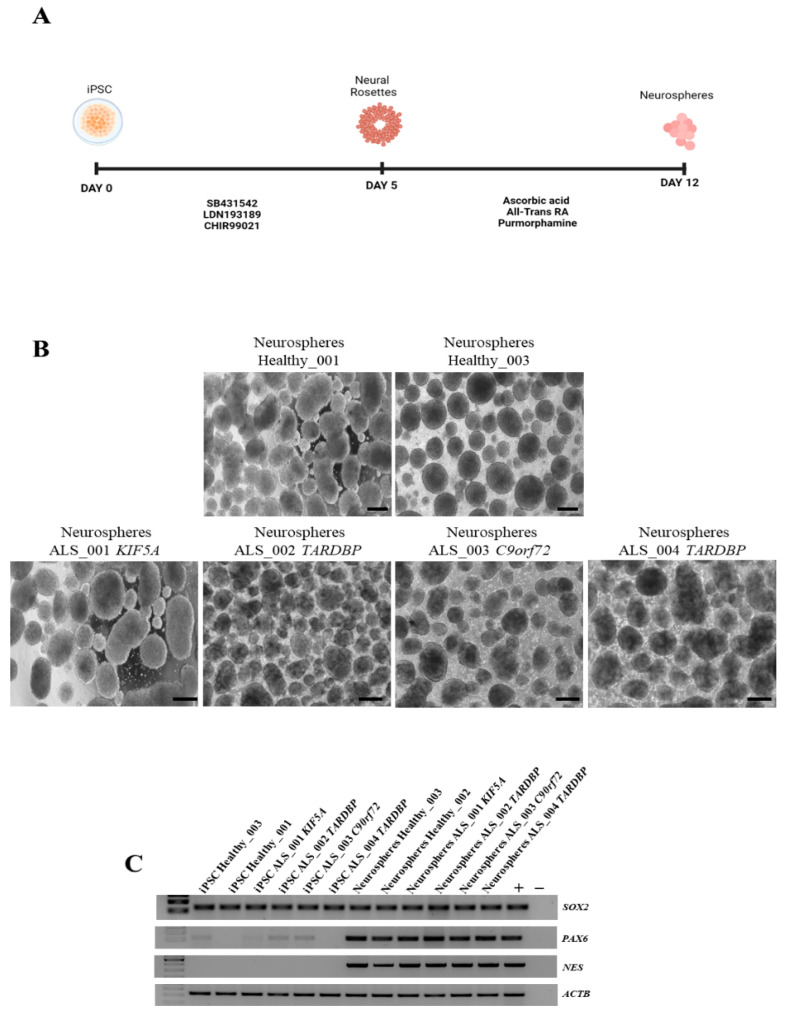
Neurosphere generation and characterization. (**A**) Schematic representation of neurosphere differentiation. (**B**) Representative images of neurospheres generated from healthy and ALS iPSCs. Scale bar, 100 μm. (**C**) RT-PCR for the expression of the neuroectoderm markers. Hela and iPSC were used as positive control. All data are representative of three independent experiments.

Envisioning future applications of obtained iPSCs in disease modeling, we investigated their ability to differentiate into MNs and their commitment to the neuroectodermal lineage. Therefore, we induced neurosphere differentiation on two healthy iPSCs and in all ALS iPSCs (Figure 3A,B). The results showed that both healthy and ALS iPSCs were successfully differentiated into neurospheres, expressing high levels of *NES*, *PAX6*, and *SOX2* markers (Figure 3C), indicating the successful initiation of neural differentiation and the potential of these iPSC lines for further differentiation into MNs. Moreover, this differentiation step ensured that the cells had already undergone neuroectodermal commitment, making them a relevant model for subsequent transcriptomic analysis. The neurospheres obtained were therefore used for RNA sequencing to characterize their gene expression profile and gain deeper insights into their molecular identity.

### 3.4. Transcriptomic Analysis of iPSCs and Neurospheres from ALS and Healthy Controls

**Figure 4:**
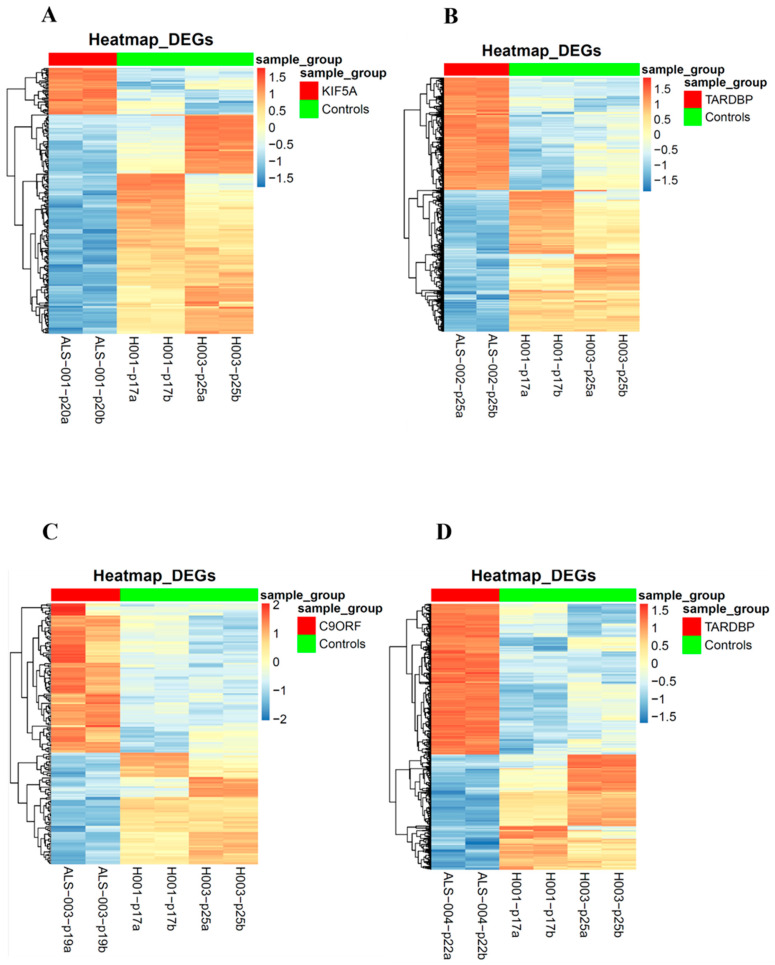
Heatmaps of the differentially expressed genes in iPSCs: Heatmaps of the differentially expressed genes of each of the four ALS patients in comparison to the matched healthy controls. Patients’ differentially expressed genes are represented on the left (top red bar); healthy controls’ differentially expressed genes are represented on the right (top green bar). (**A**): ALS001_KIF5A vs. HC; (**B**): ALS002_TARDBP vs. HC; (**C**): ALS003_c9orf72 vs. HC; (**D**): ALS004_TARDBP vs. HC.

**Figure 5:**
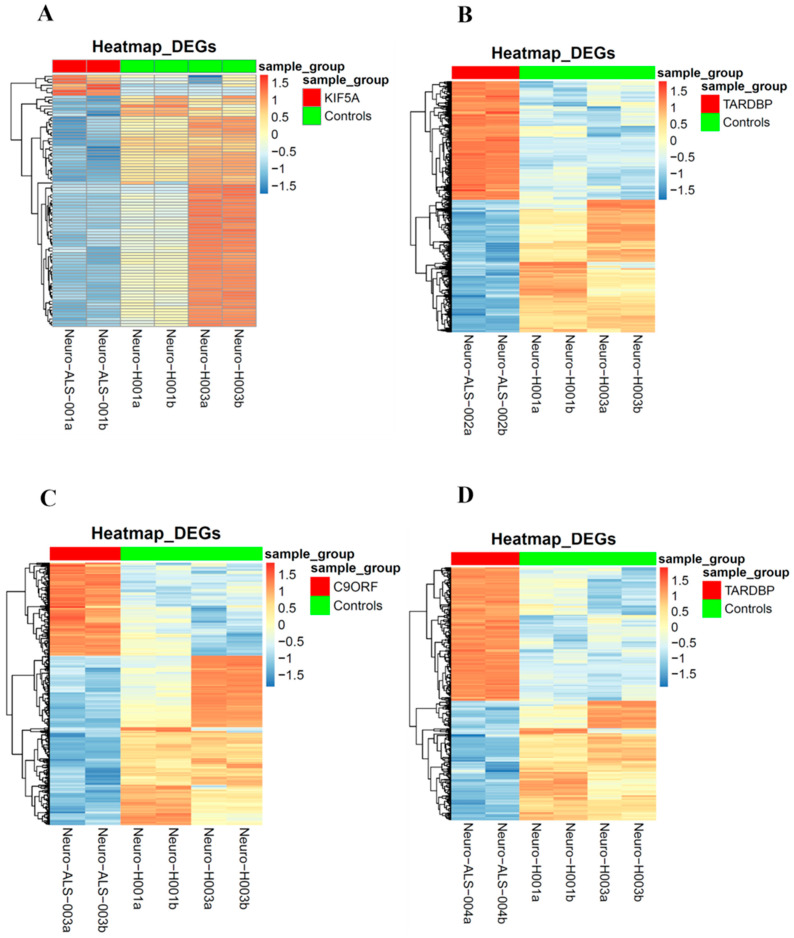
Heatmaps of the differentially expressed genes in neurospheres: Heatmaps of the differentially expressed genes of each of the four ALS patients in comparison to the matched healthy controls. Patients’ differentially expressed genes are represented on the left (top red bar); healthy controls’ differentially expressed genes are represented on the right (top green bar). (**A**): ALS001_KIF5A vs. HC; (**B**): ALS002_TARDBP vs. HC; (**C**): ALS003_c9orf72 vs. HC; (**D**): ALS004_TARDBP vs. HC.

**Figure 6:**
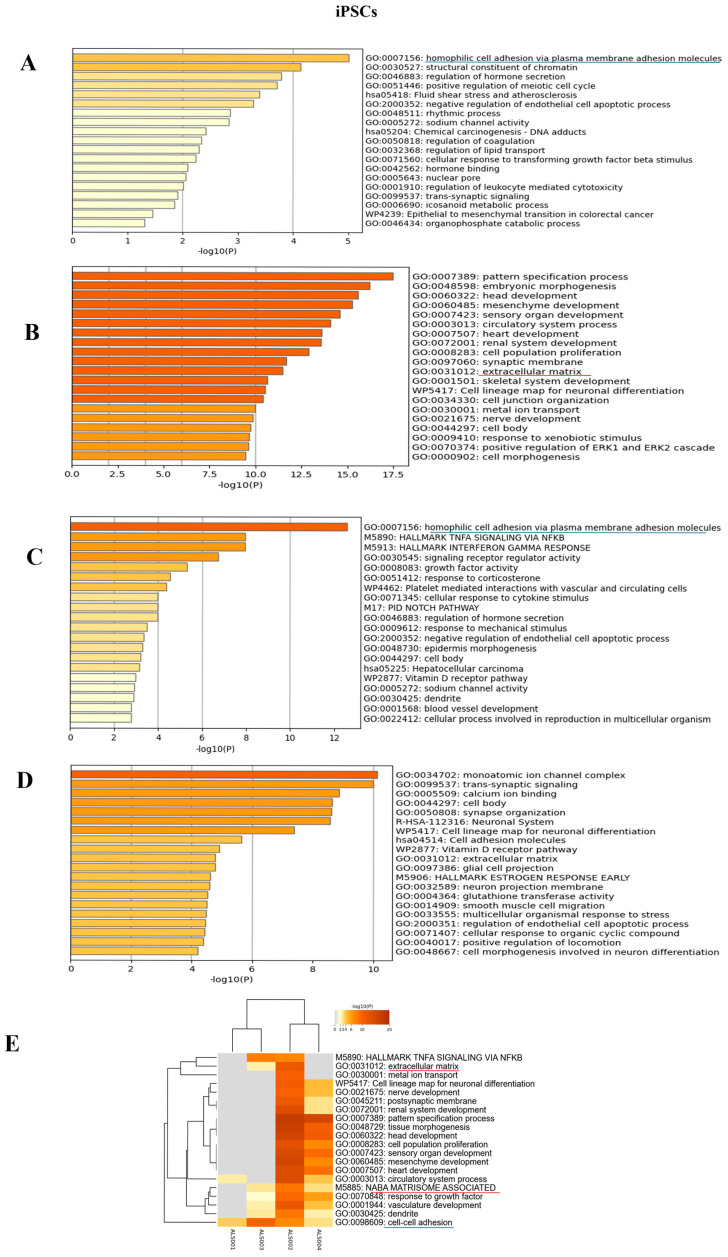
Enrichment analysis: Enrichment analysis results of the comparison between each of the four ALS patients and the matched healthy controls, at iPSC level. (**A**): Enrichment analysis for ALS001_KIF5A vs. HC; (**B**): enrichment analysis for ALS002_TARDBP vs. HC; (**C**): enrichment analysis for ALS003_c9orf72 vs. HC; (**D**): enrichment analysis results for ALS004_TARDBP vs. HC; (**E**): integrative and comparative enrichment analysis among the four subjects. The DEGs for all comparisons include either upregulated or downregulated genes. At iPSC level, we underlined in different colors the shared pathways (blue for cell-adhesion pathways and red for matrisome, extracellular-matrix pathways).

**Figure 7:**
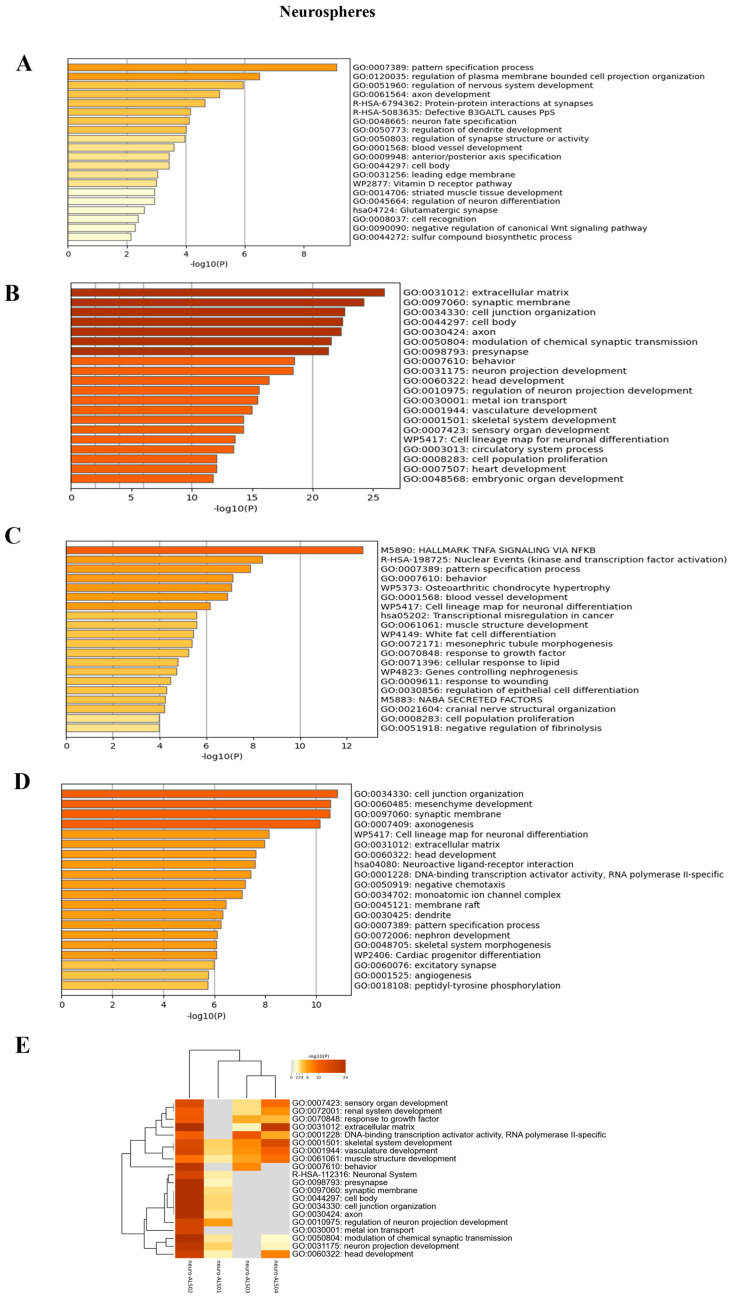
Enrichment analysis: Enrichment analysis results of the comparison between each of the four ALS patients and the matched healthy controls, at neurosphere level. (**A**): Enrichment analysis for ALS001_KIF5A vs. HC; (**B**): enrichment analysis for ALS002_TARDBP vs. HC; (**C**): enrichment analysis for ALS003_c9orf72 vs. HC; (**D**): enrichment analysis results for ALS004_TARDBP vs. HC; (**E**): integrative and comparative enrichment analysis among the four subjects. The DEGs for all comparisons include either upregulated or downregulated genes.

To identify expression patterns and pathways implicated in ALS across different mutations, we performed whole-transcriptome sequencing (RNA-seq) analysis on technical duplicates from iPSCs and neurospheres, from the four patients carrying a pathogenetic mutation in one ALS gene, namely *KIF5A* (ALS_001), *TARDBP* (ALS_002 and ALS_004), and *C9orf72* (ALS_003), and two healthy gender-matched controls (H00_1 and H00_3), for a total of 24 samples. The third healthy donor (H_002) was excluded from this analysis due to the gender mismatch with ALS cases. After PCA inspection (Supplementary Figure S2), no outlier was removed, with PC1 and PC2 explaining, respectively, 61.97% and 9.77% of variability. For both iPSCs and neurospheres, the transcriptomic profile of each patient has been compared to the healthy controls (Supplementary Figures S3 and S4). By filtering for *p*-value adj ≤ 0.05 and |log2FC| > 2, when comparing each ALS iPSC with the healthy controls, we obtained 234 DEGs (41 upregulated and 194 downregulated genes) for ALS001_*KIF5A* (Figure 4A); 1001 DEGs (443 upregulated and 558 downregulated genes) for ALS002_*TARDBP* (Figure 4B); 171 DEGs (98 upregulated and 73 downregulated genes) for ALS003_*C9orf72* (Figure 4C); and 444 DEGs (252 upregulated and 192 downregulated genes) for ALS004_*TARDBP* (Figure 4D). For neurospheres, by applying the same cut-offs, we obtained 85 DEGs (7 upregulated and 78 downregulated genes) for *ALS001_KIF5A* (Figure 5A); 1334 DEGs (630 upregulated and 704 downregulated genes) for *ALS002_TARDBP* (Figure 5B); 319 DEGs (113 upregulated and 206 downregulated genes) for *ALS003_C9orf72* (Figure 5C); and 324 DEGs (171 upregulated and 153 downregulated genes) for *ALS004_TARDBP* (Figure 5D). At iPSCs levels, only 10 differentially expressed genes were shared among all subjects (*HTR7*, *PCAT7*, *NAMPTP1*, *POM121B*, *MEG3*, *ABHD12B*, *MEG8*, *DLK1*, *C1DP1*, and *CCDC144NL-AS1*), while for neurospheres we identified 2 common DEGs (*PDZRN4* and *SLC35F4*) downregulated in all subjects. Subsequently, for each comparison an enrichment analysis by Metascape was performed on the totality of DEGs. Most of the enriched pathways could be observed only in single comparisons, and no common GO terms were found across all four patients for both iPSCs and neurospheres (Figure 6A–D and Figure 7A–D). However, an integrative and comparative analysis highlighted that some GO terms were shared at least by three subjects at the iPSC level (Figure 6E, Supplementary Figure S5, Supplementary Table S1); for example, homophilic cell adhesion via plasma membrane adhesion molecules (GO:0007156), shared by ALS001_*KIF5A*, ALS003_*C9orf72*, and ALS004_*TARDBP* (Figure 6A–D); cell–cell adhesion (GO:0098609), shared by ALS001*_KIF5A*, ALS002*_TARDBP*, ALS003*_C9orf72*, and ALS004*_TARDBP* (Figure 6E). Interestingly, we also found an enrichment in the GO terms related to the extracellular matrix (ECM), including the Naba matrisome-associated (M5885), shared by ALS002*_TARDBP*, ALS003*_C9orf72* and ALS004*_TARDBP*, and extracellular matrix (GO:0031012) in ALS002_*TARDBP* and ALS003_*C9orf72*, suggesting a general role of ECM in ALS pathological process, independently of the mutation (Figure 6E). A deeper inspection of the matrisome GO term revealed that four genes (*FGF4*, *SCG2*, *GDF15*, and *EDN1*) were shared among DEGs identified in at least three subjects, maintaining the same upregulated or downregulated trend in all the comparisons. Among the DEGs enriched in both the cell adhesion-related pathways (GO:0007156 and GO:0098609), we also highlighted several genes belonging to the protocadherin family, which are already known to be involved in disease process [22,23] and organized in a specific hub when studying the molecular interaction of ALS001_KIF5A DEGs by network analysis using String (Supplementary Figure S6). Regarding the transcriptomic profile of neurospheres, we could not identify pathways shared by at least three subjects. Indeed, most of the enriched pathways were specific to each subject carrying a different mutation and reflected the individual disease course (Figure 7A–D). Most similarities have been highlighted in the two TARDP patients (ALS002_*TARDBP* and ALS004_*TARDBP*) and were associated with GO terms linked to the extracellular matrix (Figure 7E). Generally, in all subjects the most enriched pathways at the neurosphere level reflected neuronal processes, such as axon development (GO:0061564), regulation of dendrite development (GO:0050773), synaptic membrane (GO:0097060), and neuron projection development (GO:0031175) (Figure 7A–D). 

To better understand the pathways specifically associated with the different mutations, we conducted enrichment analyses using Metascape, focusing separately on upregulated and downregulated genes for each of the four ALS patients (Supplementary Figures S8 and S9). In general, the enrichment analysis of upregulated genes revealed processes related to the known or potential functions of the mutated genes, both at the iPSC and neurosphere levels. Conversely, in neurospheres, we observed that the majority of neuronal-related pathways were consistently downregulated across all subjects (Supplementary Figure S9).

## 4. Discussion

ALS belongs to the class of neurodegenerative diseases characterized by the loss of neuronal function. ALS preferentially affects upper and lower MNs and can be classified into familial (fALS) and sporadic (sALS) ALS, with genetic mutations identified in over 40 genes including *SOD1*, *C9orf72*, *FUS*, *TARDBP*, and *KIF5A*. These mutations interfere with multiple cellular processes, such as nucleocytoplasmic transport and axonal and vesicular transport. In this study, we utilized iPSCs and neurospheres derived from four ALS patients carrying mutations in the *KIF5A*, *TARDBP*, and *C9orf72* genes, as well as from two healthy donors, to investigate transcriptomic differences associated with these ALS-related genetic mutations and to identify the corresponding molecular pathways. By differentiating iPSCs into neurospheres, we explored their potential to become MNs. Small molecules enhanced the efficient and specific conversion of both healthy and ALS iPSCs into neurospheres expressing the typical markers NES, PAX6, and SOX2. The transcriptomic profile of all ALS iPSCs was compared to two healthy gender-matched controls (H_001 and H003) showing that only 10 differentially expressed genes (DEGs) were shared across ALS iPSCs, highlighting the wide heterogeneity of ALS pathogenesis. Interestingly, among these genes, *POM121B* was downregulated in all ALS iPSCs. This gene encodes for a transmembrane protein essential for nuclear pore complex assembly and nucleocytoplasmic transport of RNAs and proteins [24]. A nuclear depletion of *POM121B* has been observed in iPSC-derived neurons and postmortem tissue from *C9orf72* patients [25] and *TARDBP* iPSC-derived MNs [26], suggesting its key role as an early pathogenic event and in the nuclear pore complex disruption. Intriguingly, *POM121B* was downregulated also in *KIF5A* iPSCs, indicating a potential link between axonal transport defects and impairment in nucleocytoplasmic transport [27]. Considering that nucleocytoplasmic transport defects are a common hallmark of neurodegenerative diseases, future studies aiming to examine the nuclear protein complex expression in ALS may aid in the identification of a potential therapeutic target. By analyzing the common GO terms in ALS iPSCs, we found an enrichment in GO terms related to matrisome-associated terms as the extracellular matrix (GO:0031012) [28,29], cell–cell adhesion (“homophilic cell adhesion via plasma membrane adhesion molecules” (GO:0007156), “cell adhesion via plasma membrane adhesion molecules” (GO:0098609), and a modulation of the Notch signaling pathway, highlighting its potential role in ALS pathophysiology. Indeed, the Notch pathway regulates critical processes such as cell differentiation, survival, and neuroinflammation, all of which are disrupted in ALS [30,31]. 

Moreover, altered cell adhesion molecule (CAM) expression suggests a dysfunction that impairs neuronal differentiation, neuronal maturation, and synaptic organization [32] and promotes immune infiltration into CNS, contributing to neuroinflammation [33]. Finally, CAM closely collaborates with the ECM, regulating processes that are fundamental for the homeostasis of nervous tissue. Among ECM genes and NABA pathways, *GDF15* overexpression is of great interest given its emerging role as an ALS biomarker [34]. Considering that unprocessed *GDF15* remains bound to the ECM, this could suggest that its upregulation may act as a compensatory mechanism [35]. On the other hand, as a stress-responsive cytokine, *GDF15* may exert a protective effect by counteracting neuroinflammation, metabolic alterations, and ECM remodeling, thus mitigating neuronal damage. 

Interestingly, an enrichment in GO terms in ALS iPSCs was directly correlated with the function of the mutated protein. Indeed, in ALS_001 *KIF5A* iPSCs, upregulated genes were enriched for the “transport vesicle membrane” (GO:00306580133), directly connected to *KIF5A* function [36]. In both *TARDBP* iPSCs, the “DNA-binding transcription factor activity” (GO:0001228) was enriched [34], reflecting the role of TDP-43 protein in transcription and splicing [37]. Finally, ALS _003 *C9orf72* iPSCs showed an enrichment in several pathways linked to immunity and inflammation (M5913 “hallmark interferon gamma response”, M5890 “TNFα signalling via NF-κB”, and WP619 “Type II interferon signalling”), underlining the specific role of *C9orf72* in eliciting immune activity and regulation highlighting the role of inflammation in neurodegeneration [38,39]. 

We further investigated whether transcriptomic differences also occur at neurosphere level. Interestingly, in iPSC-derived neurospheres no common pathways were shared across all samples, suggesting that the molecular signature could reflect the distinct molecular mechanisms driven by the different ALS-related mutations. In the two *TARDBP* neurospheres, a significant upregulation of the ECM pathway was observed, suggesting that these alterations may influence cellular behavior, tissue remodeling, and neuroinflammation. Indeed, in ALS, excessive ECM remodeling could promote fibrosis, impair neuron–glia communication, and hinder neuro regeneration, ultimately exacerbating neurodegeneration [40]. A detailed analysis of the specific ECM components affected, such as collagen, laminins, or proteoglycans, could provide insights into disease progression and help identify novel therapeutic strategies.

Moreover, an enrichment of several GO terms related to key neuronal processes, such as axon development, dendrite regulation, synaptic membrane formation, and neuron projection development were found, suggesting that the disruption in these fundamental mechanisms might occur both from pathological changes in mature MNs and from early defects in differentiation and neuron network formation [41,42]. Indeed, the disruption of these processes may lead to impaired neuronal circuit formation, reduced synaptic plasticity, and deficits in neural communication, all of which are hallmarks of ALS pathology. 

In conclusion, this study highlights the advantages of iPSC technology in ALS research by providing a renewable source of patient-specific cells to investigate the complex molecular landscape of ALS. The next step should focus on differentiating these iPSCs into MNs and other cell types to develop organoids containing ALS-derived cells, performing comprehensive transcriptomic and proteomic analyses, and investigating the functional consequences of ALS-associated mutations. Moreover, our study is perfectly in line with other international initiatives, such as Answer ALS, a large-scale resource for sporadic and familial ALS in the United States combining clinical and multi-omics data from iPSCs and iPSC-derived MNs [43]. Although our work includes iPSCs derived from patients carrying different mutations, thus contributing to increases in the diversity and representativeness of the available cellular models, it shows some limitations. The major concerns are the small sample size and limited number of control cell lines. For these reasons, this work should be considered as a pilot study. To strengthen the obtained results, future studies including a larger cohort and a higher number of independent lines for each genotype should be used. Given the urgent need for effective treatments for ALS, this study represents a step forward in leveraging stem cell technology and ALS study. The integration of iPSCs with advanced transcriptomic analyses holds promise for uncovering the intricate network of pathways involved in ALS, ultimately leading to novel therapeutic strategies.

## Figures and Tables

**Table 1:** Demographic, genetic, and clinical features of included fALS patients. All patients were of Caucasian ethnicity. Cognitive status is classified based on the Strong criteria. UMN: upper motor neuron; ALS-ci: ALS-cognitive impairment. * Disease course complicated by adrenal cancer and prolonged bed rest.

Subject	Mutation	Age at Disease Onset	Age at Diagnosis	Gender	Family History	Phenotype	Cognition	Age at Death	Disease Duration (Months from Onset to Death)
ALS_001	*KIF5A* (exon 27 3’ splice junction variant, c.3020+1G>A)	61	62	Male	Mother, maternal grandfather, great-grandfather, and three first degree cousins	Prevalent UMN (spinal)	ALS-ci	66	60 *
ALS_002	*TARDBP* (c.1144G>A, p.A382T)	63	64	Male	Brother	Prevalent UMN (bulbar)	ALS-ci	67	50
ALS_003	*C9orf72* (intron 1 GGGCC repeat pathogenetic expansion)	57	58	Male	Father	Classic (spinal)	ALS-ci	60	30
ALS_004	*TARDBP* (c.1144G>A, p.A382T)	61	65	Male	Father	Prevalent UMN (spinal)	Normal	-	84 (alive)
H_001	-	43	-	Male	-	-	-	-	-
H_002	-	58	-	Female	-	-	-	-	-
H_003	-	60	-	Male	-	-	-	-	-

## Data Availability

Due to ethical and privacy constraints related to patient consent, the RNA-seq data cannot be deposited in a public repository. However, anonymized data may be made available by the corresponding author upon reasonable request and pending appropriate institutional approvals.

## Supplementary Materials

The following supporting information can be downloaded at: https://www.mdpi.com/article/10.3390/cells14130958/s1.
