# Supplementary material for: Identification of Transcriptomic Differences in Induced Pluripotent Stem Cells and Neural Progenitors from Amyotrophic Lateral Sclerosis Patients Carrying Different Mutations: A Pilot Study
Source: Cells. 2025 Jun 23;14(13):958. doi: 10.3390/cells14130958 (PMC12249345; doi:10.3390/cells14130958)
Supplement: Supplementary file 1 [file cells-14-00958-s001.zip › cells-3563933-supplementary.pdf]

Supplementary Material Sgromo et al.

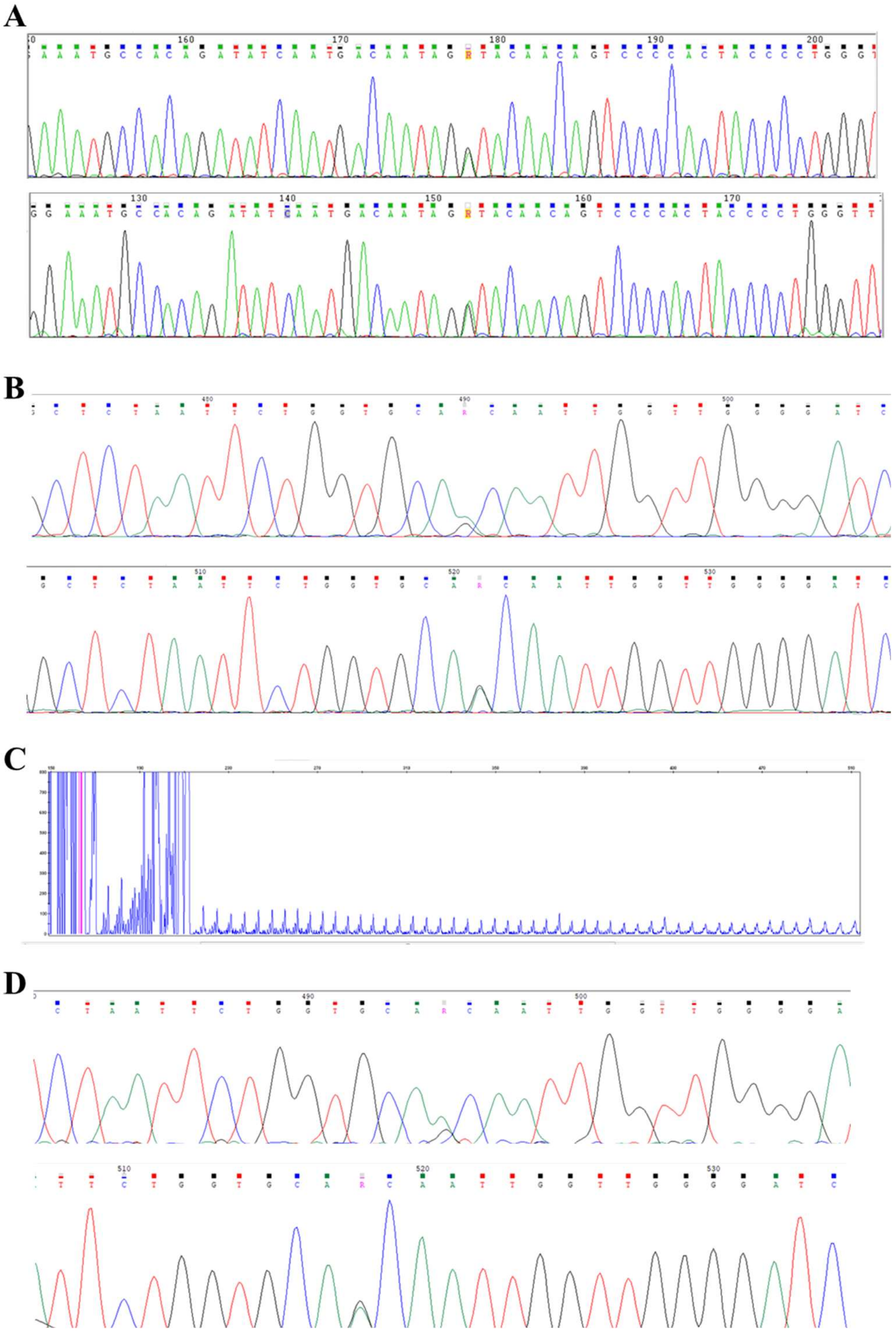

Supplementary Figure S1. Validation of the occurrence of the mutation identified in the ALS patients from in the derived iPSCs.

A) *KIF5A* (exon27 3′ splice junction variant, c.3020+1G>A, NM_004984: chr12:57582630 G>A, hg38, ALS_001). **B and D)** *TARDBP* (p. Ala382Thr, c.1144G>A, NM_007375.4, chr1-11022553 G>A , hg38 ALS_002 and ALS_004), **C)** *C9orf72* (intron 1 GGGGCC repeat pathogenetic expansion, ALS_003). Electropherograms on ABI PRISM 3130XL from Sanger sequencing (A,B,D) and PCR-tandem repeat (C).

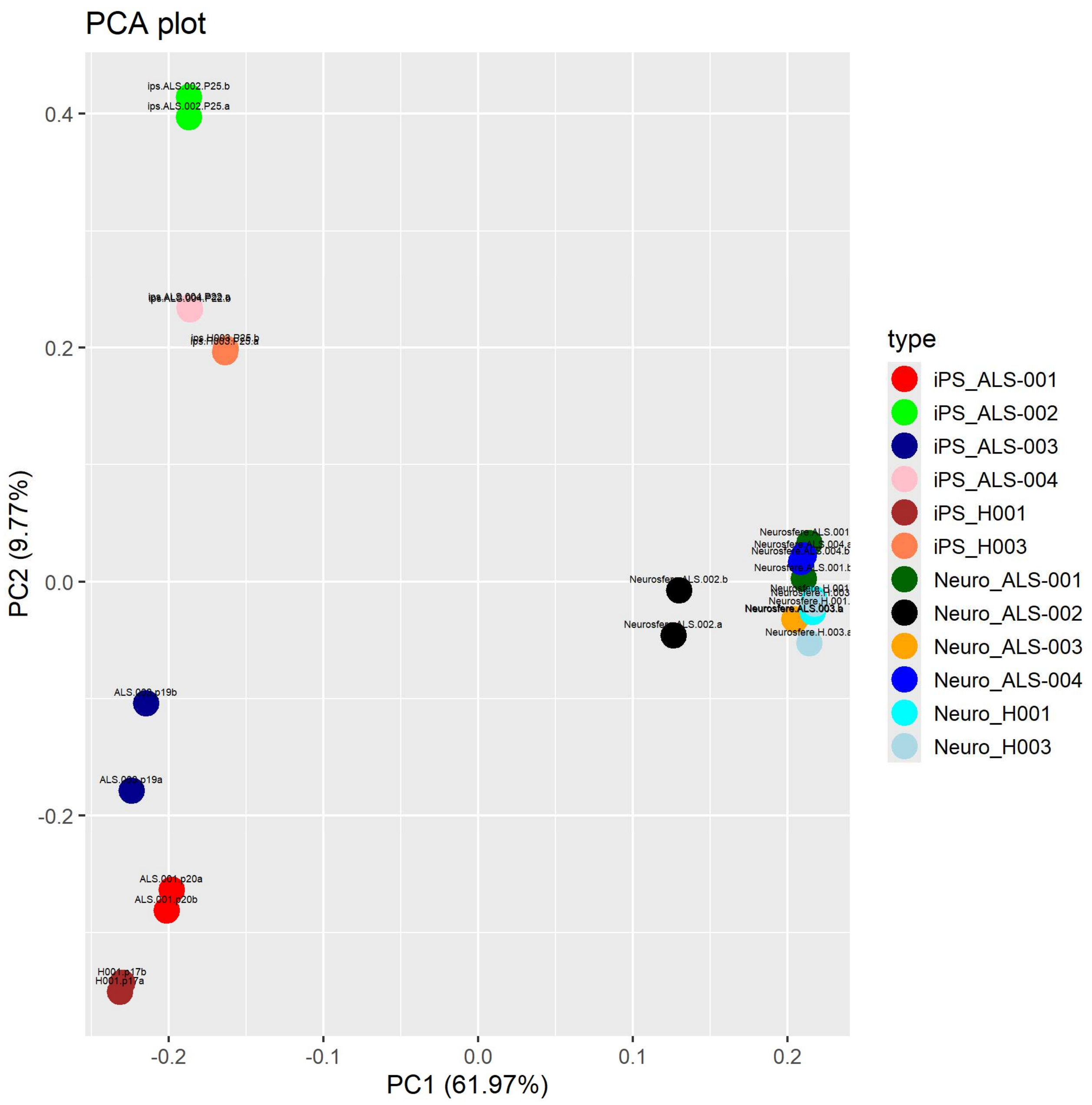

Supplementary Figure S2. Principal Component Analysis (PCA).
PCA shows the distribution of samples according to PC1 (x-axis) and PC2 (y-axis) explaining 61,97% and 9,77 %, respectively, of variability. Each dot represents a sample.

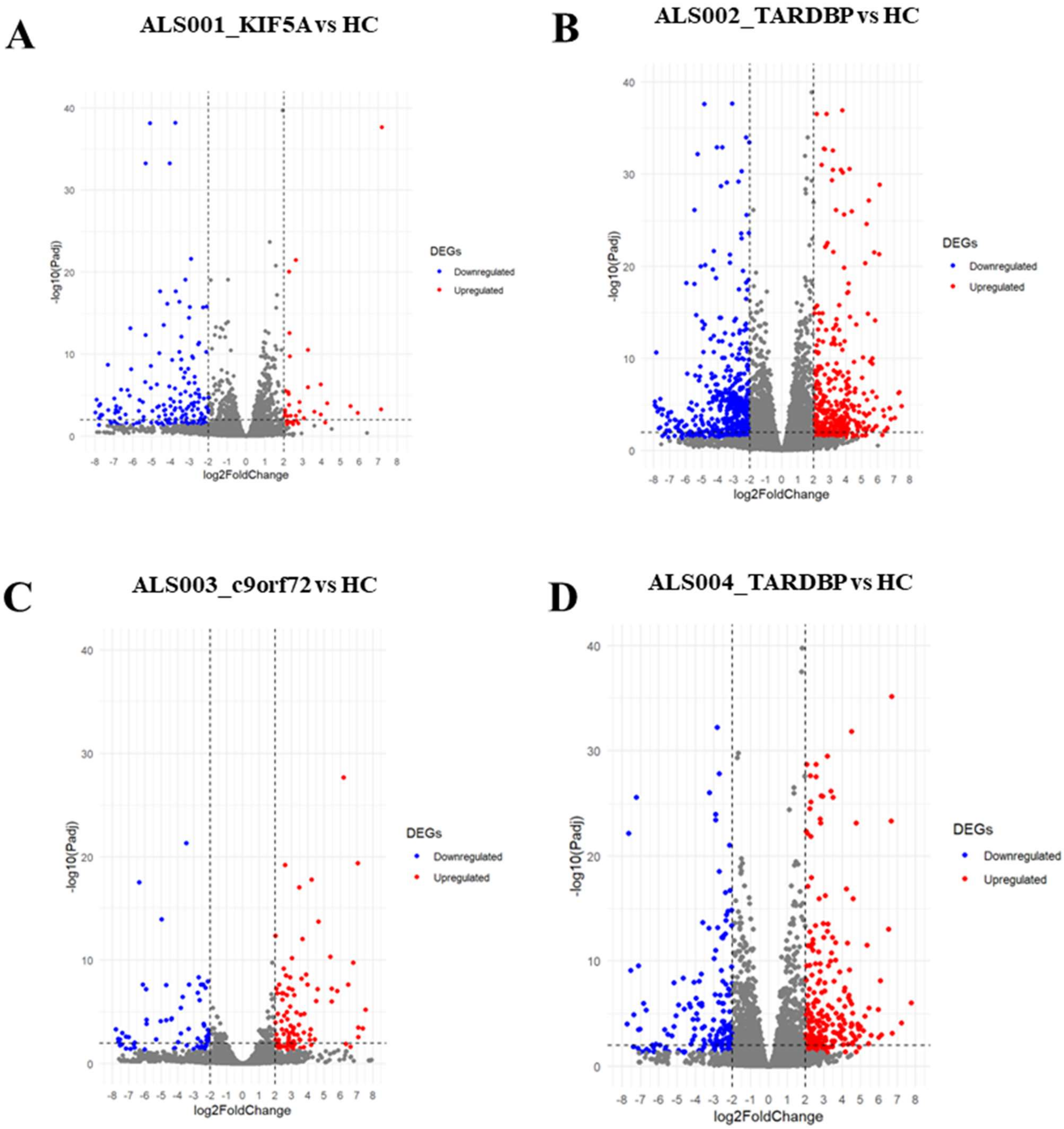

Supplementary Figure S3. Volcano Plots for the differentially expressed genes (DEGs) distribution in the four patients at iPSCs level.
A) ALS001_*KIF5A*; **B)** ALS002_*TARDBP*; **C)** ALS003_*C9orf72*; **D)** ALS004_*TARDBP*. On the x-axis the log2FC is represented, while on the y-axis -log10(p-value adjusted). Downregulated genes (log2FC < -2; p.adj < 0.05) are represented by blue dots and upregulated genes (log2FC > 2; p.adj < 0.05) by red dots.

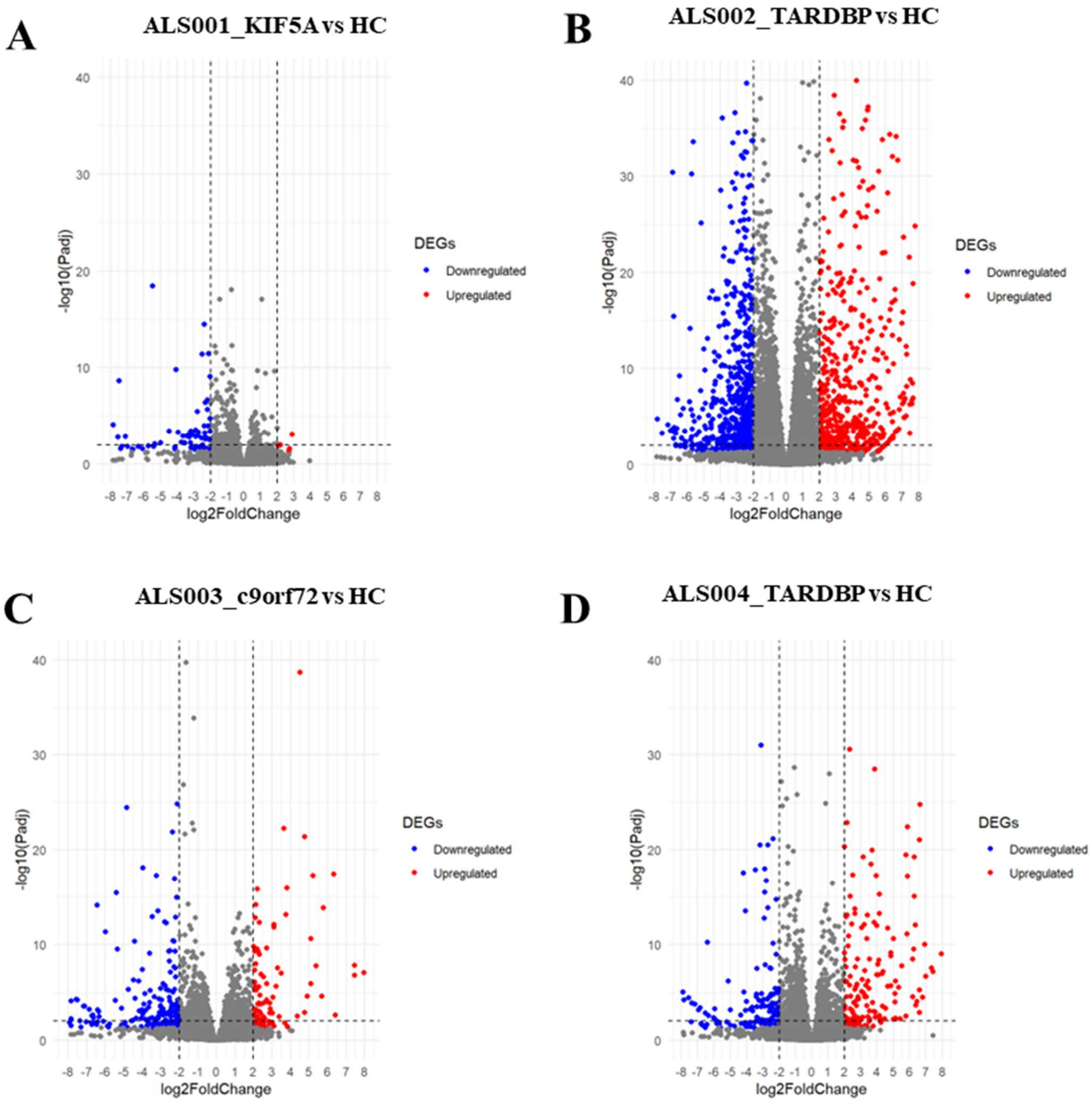

Supplementary Figure S4. Volcano Plots for the differentially expressed genes (DEGs) distribution in the four patients at neurosphere level.

A) ALS001_*KIF5A*; **B)** ALS002_*TARDBP*; **C)** ALS003_*C9orf72*; **D)** ALS004_*TARDBP*. On the x-axis the log2FC is represented, while on the y-axis -log10(p-value adjusted). Downregulated genes (log2FC < -2; p.adj < 0.05) are represented by blue dots and upregulated genes (log2FC > 2; p.adj < 0.05) by red dots.

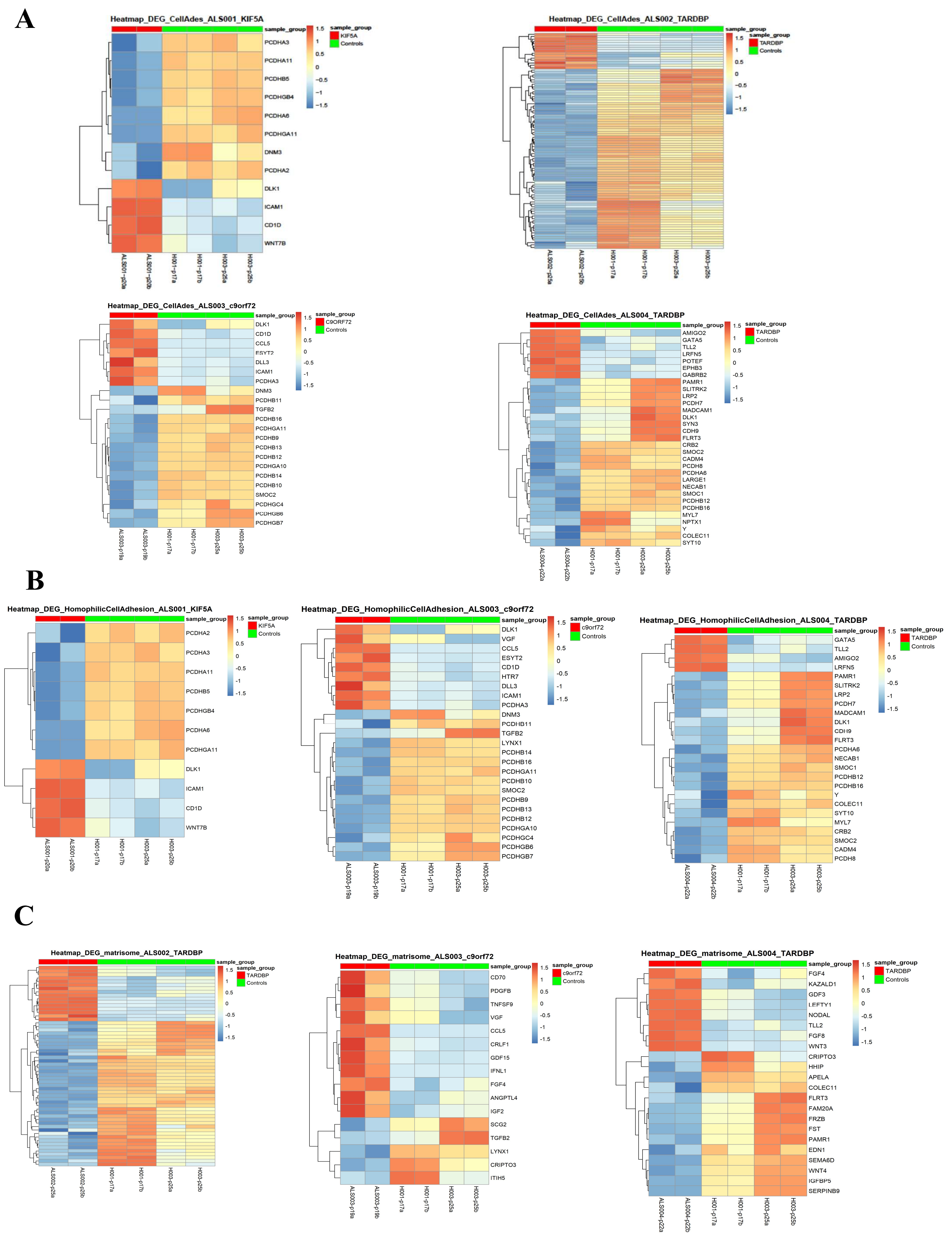
A
Heatmap_DEG_CellAdes_ALS001_KIF5A
sample_group
KIF5A
Controls
PCDHA3
PCDHA11
PCDHB5
PCDHGB4
PCDHA6
PCDHGA11
DNM3
PCDHA2
DLK1
ICAM1
CD1D
WNT7B
ALS001-p20a
ALS001-p20b
H001-p17a
H001-p17b
H003-p25a
H003-p25b
Heatmap_DEG_CellAdes_ALS002_TARDBP
TARDBP
ALS002-p25a
ALS002-p25b
Heatmap_DEG_CellAdes_ALS003_c9orf72
C9ORF72
DLK1
CD1D
CCL5
ESYT2
DLL3
ICAM1
PCDHA3
DNM3
PCDHB11
TGFB2
PCDHB16
PCDHGA11
PCDHB9
PCDHB13
PCDHB12
PCDHGA10
PCDHB14
PCDHB10
SMOC2
PCDHGC4
PCDHGB6
PCDHGB7
ALS003-p19a
ALS003-p19b
Heatmap_DEG_CellAdes_ALS004_TARDBP
AMIGO2
GATA5
TLL2
LRFN5
POTEF
EPHB3
GABRB2
PAMR1
SLITRK2
LRP2
PCDH7
MADCAM1
DLK1
SYN3
CDH9
FLRT3
CRB2
SMOC2
CADM4
PCDH8
PCDHA6
LARGE1
NECAB1
SMOC1
PCDHB12
PCDHB16
MYL7
NPTX1
Y
COLEC11
SYT10
ALS004-p22a
ALS004-p22b
B
Heatmap_DEG_HomophilicCellAdhesion_ALS001_KIF5A
Heatmap_DEG_HomophilicCellAdhesion_ALS003_c9orf72
HTR7
LYNX1
Heatmap_DEG_HomophilicCellAdhesion_ALS004_TARDBP
C
Heatmap_DEG_matrisome_ALS002_TARDBP
Heatmap_DEG_matrisome_ALS003_c9orf72
CD70
PDGFB
TNFSF9
VGF
CCL5
CRLF1
GDF15
IFNL1
FGF4
ANGPTL4
IGF2
SCG2
TGFB2
LYNX1
CRIPTO3
ITIH5
Heatmap_DEG_matrisome_ALS004_TARDBP
FGF4
KAZALD1
GDF3
LEFTY1
NODAL
TLL2
FGF8
WNT3
CRIPTO3
HHIP
APELA
COLEC11
FLRT3
FAM20A
FRZB
FST
PAMR1
EDN1
SEMA6D
WNT4
IGFBP5
SERPINB9

Supplementary Figure S5. Heatmap of DEGs belonging to the pathways shared among at least three subjects.

Heatmap of differentially expressed genes associated with each GO term listed in Supplementary Table 1 for specific subjects. **A)** A) Cell-cell adhesion (GO:0098609): ALS001_*KIF5A*, ALS002_*TARDBP*, ALS003_*C9orf72*, ALS004_*TARDBP*; B) Homophilic cell adhesion via plasma membrane adhesion molecules (GO:0007156): ALS001_*KIF5A*, ALS003_*C9orf72*, ALS004_*TARDBP*; C) Naba Matrisome Associated (M5885): ALS002_*TARDBP*, ALS003_*C9orf72*, ALS004_*TARDBP*.

iPSCs

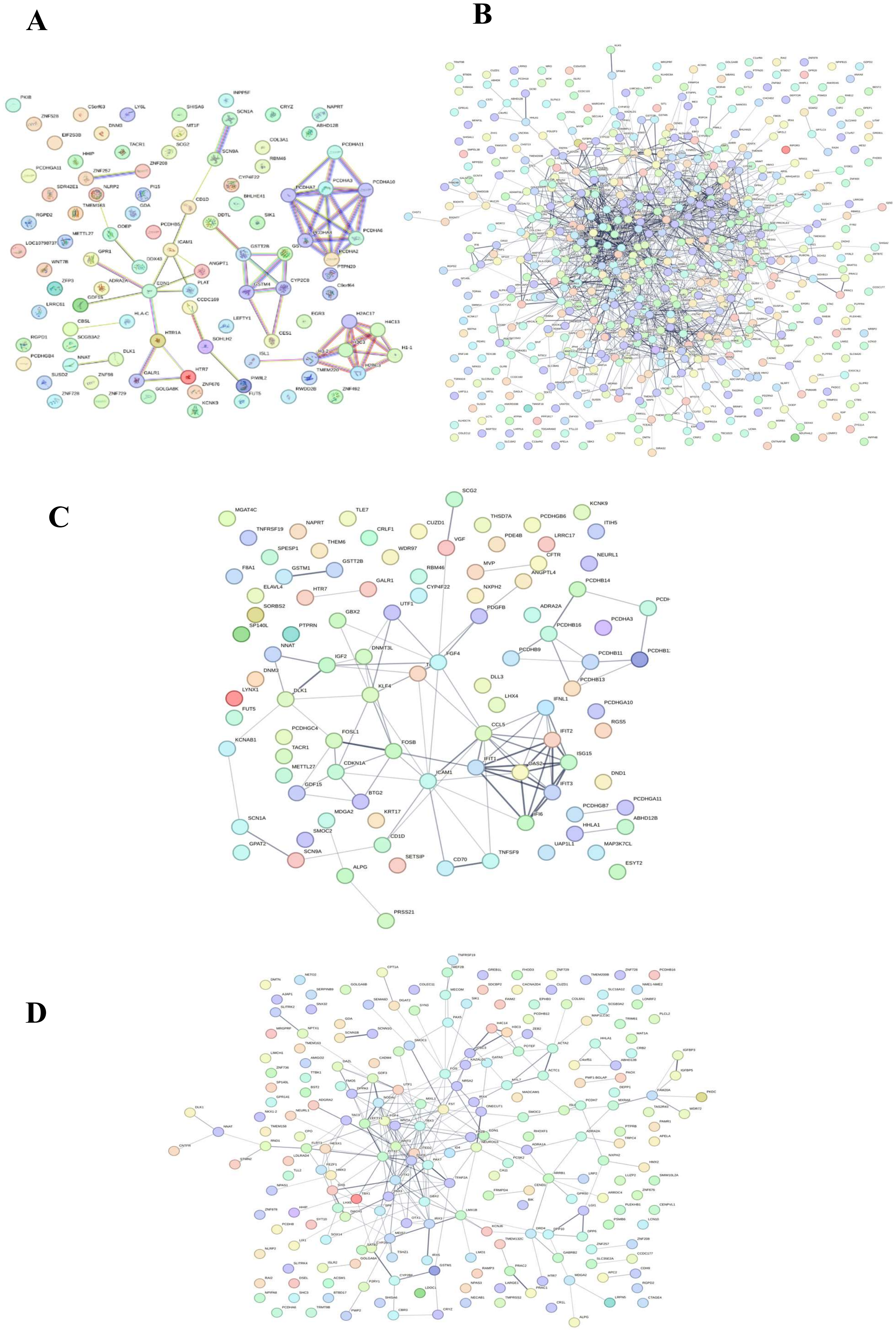

Supplementary Figure S6. Network analysis of DEGs.
Molecular interaction analyses of DEGs obtained for each patient at iPSCs-level produced by String.
A) ALS001_*KIF5A*; **B)** ALS002_*TARDBP*; **C)** ALS003_*C9orf72*; **D)** ALS004_*TARDBP*.

Neurospheres

A

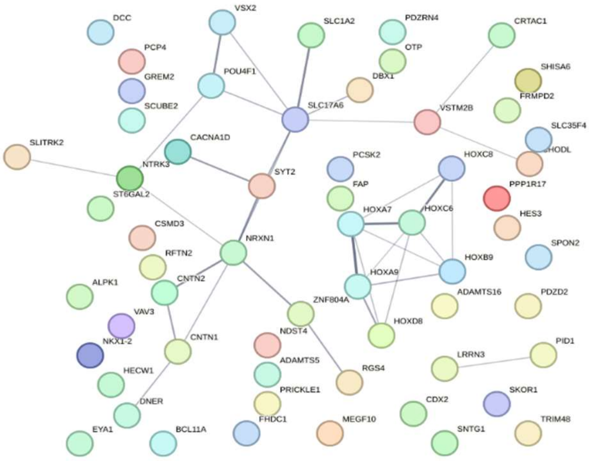

B

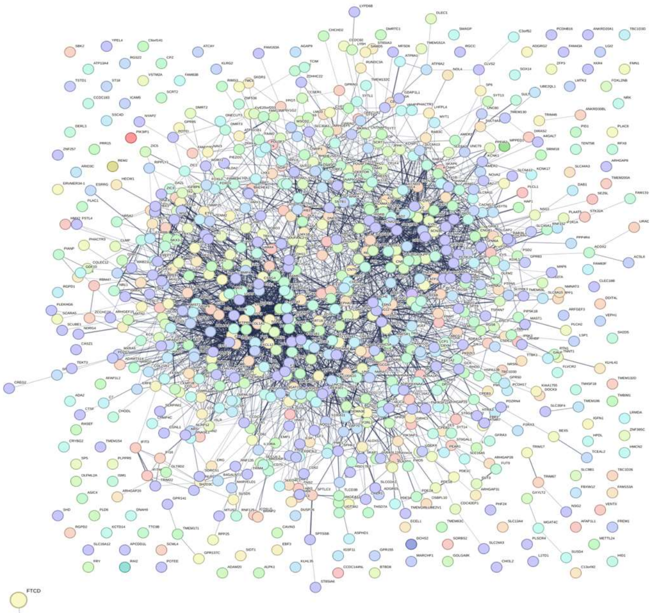

C

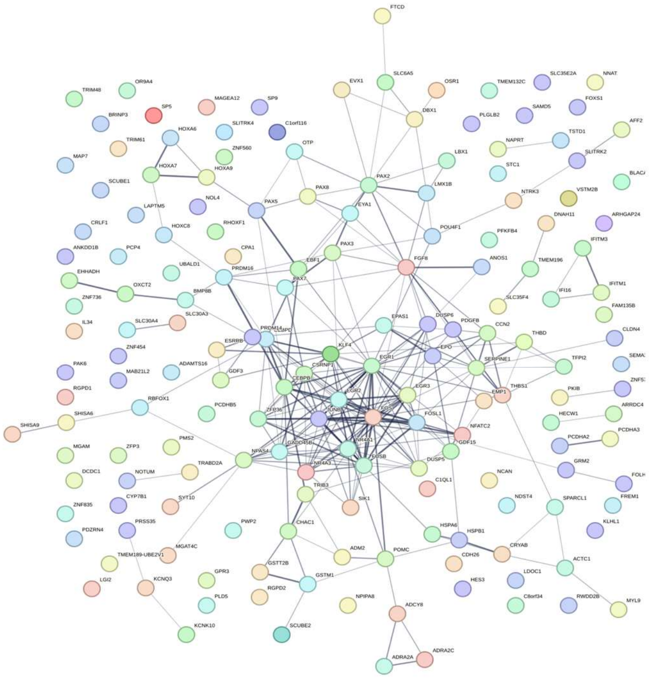

D

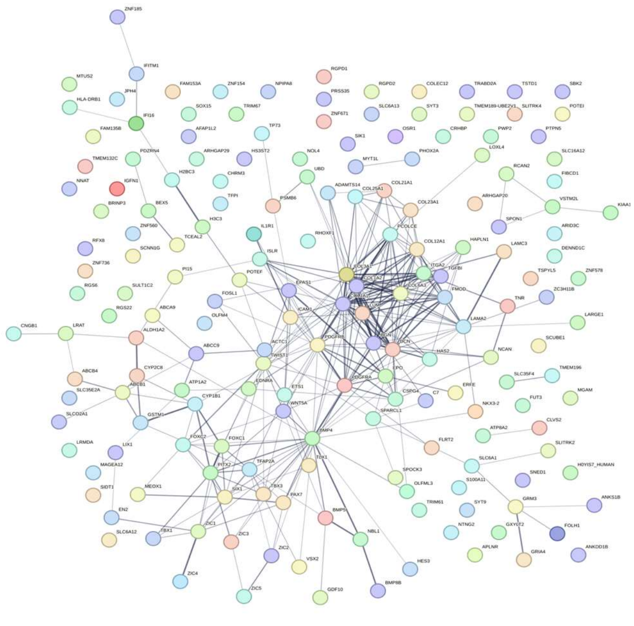

Supplementary Figure S7. Network analysis of DEGs.
Molecular interaction analyses of DEGs obtained for each patient at neurosphere level produced by String. **A)** ALS001_*KIF5A*; **B)** ALS002_*TARDBP*; **C)** ALS003_*C9orf72*; **D)** ALS004_*TARDBP*.

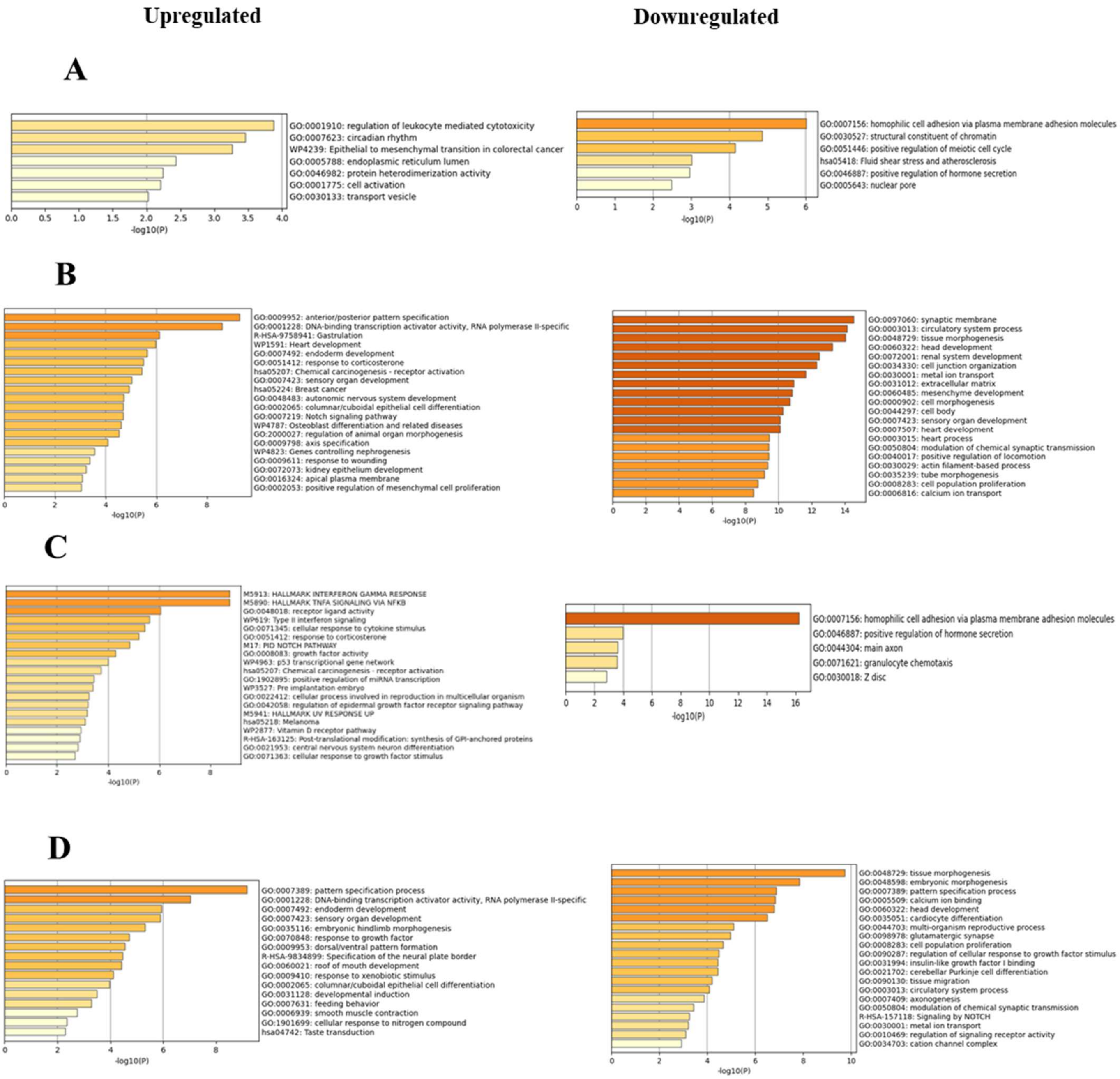

Supplementary Figure S8. Enrichment analyses.

Enrichment analyses produced by Metascape and conducted separately on upregulated (on the right) and downregulated (on the left) genes on DEGs obtained for each patient at iPSCs level. **A)** ALS001_*KIF5A*; **B)** ALS002_*TARDBP*; **C)** ALS003_*C9orf72*; **D)** ALS004_*TARDBP*.

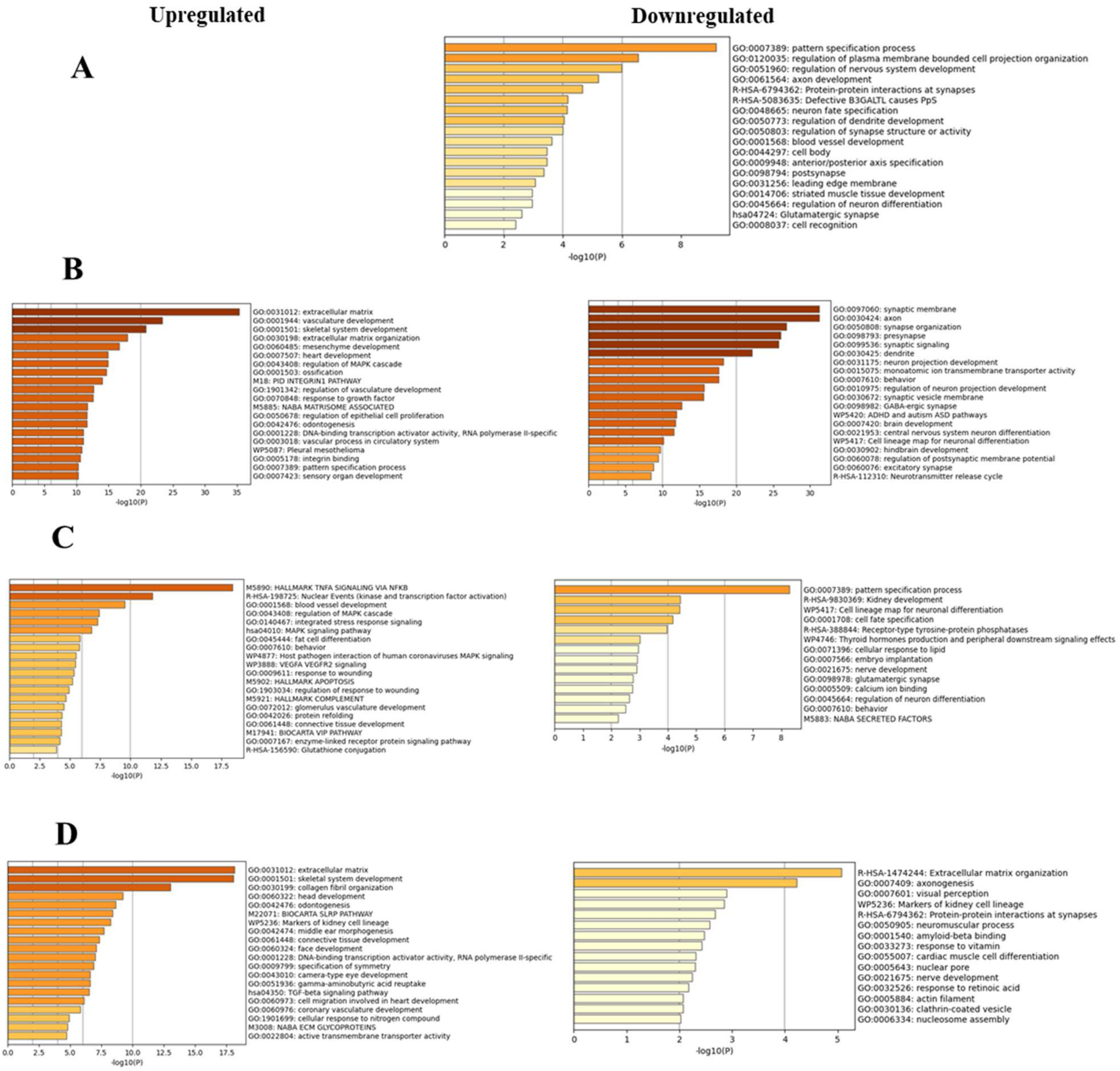

Supplementary Figure S9. Enrichment analyses.

Enrichment analyses produced by Metascape and conducted separately on upregulated (on the right) and downregulated (on the left) genes on DEGs obtained for each patient at neurosphere level. **A)** ALS001_*KIF5A*; **B)** ALS002_*TARDBP*; **C)** ALS003_*C9orf72*; **D)** ALS004_*TARDBP*.

Supplementary Table S1. DEGs belonging to the three pathways shared among at least three subjects at iPSCs level. Differentially expressed genes belonging to the specific pathway for each subject are listed.

Ontology	Genes	Patient
Cell-cell adhesion (GO:0098609)	*DLK1, CD1D, PCDHA3, PCDHGA11, DNM3, PCDHA6, ICAM1, PCDHA2, PCDHGB4, PCDHB5, WNT7B, PCDHA11*	ALS001_KIF5A
	DLK1, CD1D, CDH9, PCDH8, LRFN5, SMOC1, LRP2, NPTX1, PCDH7, PAMR1, GATA5, CRB2, Y, COLEC11, SYT10, FLRT3, POTEF, ESYT2, DLL3, OC90, RIMS1, MYOF, CX3CL1, PTPRO, PLXNA4, NRP1, EPDR1, SLC8A1, CDHR3, LCP1, BHLHA15, LPL, GRIA1, TTN, NLGN3, PLA2G2A, CDH7, CALB1, CNTN2, VIL1, CHP2, WDR49, ITGA4, SPN, SCUBE2, PCDH18, PKP1, GJA4, NTNG1, GLRB, TNC, COL17A1, F10, EPHA4, GABRA2, DOCK10, AIF1, ASTN1, SYN1, LIMS2, LRRC4C, CSRP1, ABI3, PEAR1, DLL1, S100A4, MPZL2, PLA2G4C, MATN4, SEZ6, FBLN7, INA, LEF1, CADM2, NFIA, ARHGAP6, PDZRN3, FN1, PRICKLE1, DNER, HPCAL4, CDH6, SLC1A1, LRRC4B, CYP1B1, DCHS2, ANXA9, LRRTM1, MYL9, NRXN2	ALS002_TARDBP
	DLK1, CD1D, PCDHA3, PCDHGA11, PCDHB12, DNM3, SMOC2, ICAM1, ESYT2, PCDHB16, DLL3, TGFB2, PCDHGB7, PCDHB10, PCDHGA10, PCDHB9, PCDHGB6, PCDHB13, CCL5, PCDHB14, PCDHGC4, PCDHB11	ALS003_c9orf72
	DLK1, CDH9, PCDH8, LRFN5, SMOC1, PCDHB12, LRP2, NPTX1, PCDH7, PAMR1, GATA5, CRB2, SMOC2, Y, COLEC11, SYT10, FLRT3, PCDHA6, POTEF, PCDHB16, CADM4, AMIGO2, EPHB3, MADCAM1, GABRB2, SLITRK2, NECAB1, TLL2, LARGE1, MYL7, SYN3	ALS004_TARDBP
Homophilic cell adhesion via plasma membrane adhesion molecules (GO:0007156)	*PCDHA11, PCDHB5, ICAM1, PCDHA6, PCDHGA11, PCDHGB4, CD1D, PCDHA3, PCDHA2, WNT7B, DLK1*	ALS001_KIF5A
	PCDHGA10, LYNX1, HTR7, PCDHB16, ESYT2, PCDHGA11, PCDHB12, PCDHB13, SMOC2, ICAM1, PCDHGB7, PCDHB14, DLL3, PCDHB9, PCDHB10, PCDHA3, PCDHGB6, CCL5, PCDHGC4, CD1D, DNM3, DLK1, VGF, PCDHB11, TGFB2	ALS003_c9orf72
	CDH9, PCDH7, PCDH8, FLRT3, PCDHB12, PCDHA6, PCDHB16, SLITRK2, GATA5, LRFN5, CADM4, CRB2, AMIGO2, LRP2, TLL2, DLK1, RGN, PAMR1, MYL7, SMOC1, SMOC2, NECAB1, COLEC11, SYT10, MADCAM1	ALS004_TARDBP
Naba Matrisome Associated (M5885)	*FGF4, SCG2, GDF15, EDN1, APELA, KAZALD1, GDF3, SEMA6D, WNT3, PAMR1, NODAL, WNT4, COLEC11, FLRT3, PLAT, FGF8, FRZB, CRLF1, FGF19, AMBP, CX3CL1, PLXNA4, ENG, ANGPTL2, STC1, CST1, COLEC12, TAC1, SCUBE2, ADAM12, BMP2, MUC20, HYAL3, ADAMTS5, GPC3, F10, ADAMTS4, PDGFA, EDN3, IL23A, LYPD1, SFRP1, DLL1, S100A4, CXCL6, ADAMTS9, NRG1, WIF1, FN1, NTF3, SERPINA5, BMP6, SERPINF2, ADAMTSL2, ANXA9, SERPINE1, PAPPA, CER1*	ALS002_TARDBP

	FGF4, SCG2, GDF15, CRIPTO3, CRLF1, ITIH5, LYNX1, TGFB2, TNFSF9, CD70, PDGFB, ANGPTL4, IGF2, IFNL1, VGF, CCL5	ALS003_c9orf72
	FGF4, EDN1, APELA, KAZALD1, CRIPTO3, GDF3, SEMA6D, WNT3, PAMR1, NODAL, WNT4, COLEC11, FLRT3, LEFTY1, HHIP, FGF8, FRZB, SERPINB9, IGFBP5, FST, TLL2, FAM20A	ALS004_TARDBP

Supplementary Table S2. List of primers used

Gene	Synthetic oligonucleotide	Attempted band
OCT4	S: 5'-CGTAAGCAGAAGAGGATCACC-3'	179 bp
	A: 5'-GCTTCCTCCACCCACTTCTGC-3'	
SOX2	S: 5'-GCAGCTACAGCATGATGCAGG-3'	134 bp
	A: 5'-AGCTGGTCATGGAGTTGTACTGC-3'	
KLF-4	S: 5'-CCAGAGGAGCCCAAGCCAA-3'	130 bp
	A: 5'-CGCAGGTGTGCCTTGAGATG-3'	
C-Myc	S: 5'-CATCCAGGACTGTATGTGGAG-3'	227 bp
	A: 5'-GCGAGCTGCTGTCGTTGAG-3'	
OTX2	S: 5'-CAACACAGCCTCCACTGTGA-3'	509 bp
	A: 5'-AAACCATACCTGCACCCTCG-3'	
NES	S: 5'-CAGCGTTGGAACAGAGGTTGG-3'	388 bp
	A: 5'-TGGCACAGGTGTCTCAAGGGTAG-3'	
NCAM	S: 5'-ATGGAAACTCTATTAAAGTGAACCTG-3'	178 bp
	A: 5'-TAGACCTCATACTCAGCATTCCAGT-3'	
TBXT	S: 5'-CGGAACAATTCTCCAACCTATT-3'	357 bp
	A: 5'-GTACTGGCTGTCCACGATGTCT-3'	
TBX6	S: 5'-CTGTCGGACTCACCGGG-3'	477 bp
	A: 5'-AGAAACAAGTAGCGGGCCTC-3'	
ACTA2	S: 5'-CTGTTCCAGCCATCCTTCAT-3'	316 bp
	A: 5'-CGGCTTCATCGTATTCCTGT-3'	
AFP	S: 5'-ACTCCAGTAAACCCTGGTGTTG-3'	255 bp
	A: 5'-GAAATCTGCAATGACAGCCTCA-3'	
SOX17	S: 5'-CCCCAACTACAAGTACCGGC-3'	498 bp
	A: 5'-CCGTAGTACACGTGAAGGGC-3'	
FOXA2	S: 5'-ATGCACTCGGCTTCCAGTAT-3'	577 bp
	A: 5'-GGTAGATCTCGCTCAGCGTC-3'	
PAX6	S: 5'-AACGATAACATACCAAGCGTGT-3'	120 bp
	A: 5'-GGTCTGCCCGTTCAACATC-3'	
ACTB	S: 5'-TGCCTGACGGCCAGGTCAT-3'	226 bp
	A: 5'-ATCTCCTTCTGCATCCTGTCGG-3'	
SeV	S: 5'-GGATCACTAGGTGATATCGAGC-3'	181bp
	A: 5'-ACC AGACAAGAGTTTAAGAGATATGTATC-3'	
Sev-*KOS*	S: 5'-ATGCACCGCTACGACGTGAGCGC-3'	528bp
	A: 5'-ACCTTGACAATCCTGATGTGG-3'	
Sev-*KL4*	S: 5'-TTCCTGCATGCCAGAGGAGCCC-3'	410bp
	A: 5'-AATGTATCGAAGGTGCTCAA -3'	
Sev-*CMyc*	S: 5'-TAACTGACTAGCAGGCTTGTCG-3'	532bp
	A: 5'-TCCACATACAGTCCTGGATGATGATG-3'	

Supplementary Table S3. List of antibodies used

Primary antibodies	Host	Reactivity	Manufacturer	Dilution	Catalog #
OCT4	Rabbit	Human	Abcam	1:100	Ab18976
SOX2	Rabbit	Human	Abcam	1:100	Ab97959
SSEA3	Rat	Human	Abcam	1:100	Ab16286
TRA1-60	Mouse	Human	BD Bioscences	1:100	560071
KIF5A	Rabbit	Human	Invitrogen	1:1000	PA1-642
TDP-43	Rabbit	Human	Invitrogen	1:1000	MA5-35273
C9orf72	Rabbit	Human	Invitrogen	1:1000	22637-1-AP
**Secondary antibodies**	**Fluorophores**	**Manufacturer**		**Dilution**	**Catalog #**
Goat anti-Rabbit	AlexaFluor 488	Invitrogen		1:500	A11008
Goat anti-Rat	AlexaFluor 488	Invitrogen		1:500	A11006
Goat anti-Mouse	Alexa Fluor 546	Invitrogen		1:500	A11003
